# A Novel Flp Reporter Mouse Shows That TRPA1 Expression Is Largely Limited to Sensory Neuron Subsets

**DOI:** 10.1523/ENEURO.0350-23.2023

**Published:** 2023-12-04

**Authors:** Mayur J. Patil, Seol-Hee Kim, Parmvir K. Bahia, Sanjay S. Nair, Teresa S. Darcey, Jailene Fiallo, Xiao Xia Zhu, Robert D. Frisina, Stephen H. Hadley, Thomas E. Taylor-Clark

**Affiliations:** 1 Molecular Pharmacology & Physiology, Morsani College of Medicine, University of South Florida, Tampa, Florida 33612; 2 Medical Engineering, College of Engineering, University of South Florida, Tampa, Florida 33620

**Keywords:** afferent, DRG, mapping, nociception, TRPA1, vagal

## Abstract

Transient receptor potential ankyrin 1 (TRPA1) is a polymodal cation channel that is activated by electrophilic irritants, oxidative stress, cold temperature, and GPCR signaling. TRPA1 expression has been primarily identified in subsets of nociceptive sensory afferents and is considered a target for future analgesics. Nevertheless, TRPA1 has been implicated in other cell types including keratinocytes, epithelium, enterochromaffin cells, endothelium, astrocytes, and CNS neurons. Here, we developed a knock-in mouse that expresses the recombinase Flp_O_ in TRPA1-expressing cells. We crossed the *TRPA1^Flp^* mouse with the *R26^ai65f^* mouse that expresses tdTomato in a Flp-sensitive manner. We found tdTomato expression correlated well with TRPA1 mRNA expression and sensitivity to TRPA1 agonists in subsets of TRPV1 (transient receptor potential vanilloid receptor type 1)-expressing neurons in the vagal ganglia and dorsal root ganglia (DRGs), although tdTomato expression efficiency was limited in DRG. We observed tdTomato-expressing afferent fibers centrally (in the medulla and spinal cord) and peripherally in the esophagus, gut, airways, bladder, and skin. Furthermore, chemogenetic activation of TRPA1-expressing nerves in the paw evoked flinching behavior. tdTomato expression was very limited in other cell types. We found tdTomato in subepithelial cells in the gut mucosa but not in enterochromaffin cells. tdTomato was also observed in supporting cells within the cochlea, but not in hair cells. Lastly, tdTomato was occasionally observed in neurons in the somatomotor cortex and the piriform area, but not in astrocytes or vascular endothelium. Thus, this novel mouse strain may be useful for mapping and manipulating TRPA1-expressing cells and deciphering the role of TRPA1 in physiological and pathophysiological processes.

## Significance Statement

The ion channel transient receptor potential ankyrin 1 (TRPA1) is activated by oxidative stress, pollutants, cold, and downstream of GPCR activation. TRPA1 is expressed on some nociceptive sensory afferent nerves, and its activation evokes pain and nocifensive reflexes. However, there is disagreement regarding the extent of TRPA1 expression, both within specific afferent subsets and in nonafferent cells. We developed a *TRPA1^Flp^* mouse that expresses Flp recombinase in TRPA1-expressing cells, allowing for their identification and manipulation. TRPA1 was reported in vagal and DRG subsets, and their chemogenetic activation in the skin evoked pain. TRPA1 was also reported in gut subepithelial cells, cochleal supporting cells, and some cortical neurons. We found no evidence of TRPA1 reporting in enterochromaffin cells, epithelium, endothelium, keratinocytes, Schwann cells, smooth muscle cells, or astrocytes.

## Introduction

Transient receptor potential ankyrin 1 (TRPA1) is a member of the TRP superfamily of plasma membrane ion channels (Wu et al., 2010). TRPA1 channels are formed by four identical subunits and conduct cations, including Na^+^ and Ca^2+^, when activated (Paulsen et al., 2015; Zhao et al., 2020). TRPA1 are polymodal channels that are activated by numerous independent mechanisms including by electrophiles and reactive oxygen species (ROS) via covalent modification of key intracellular cysteines (Hinman et al., 2006; Macpherson et al., 2007; Andersson et al., 2008; Bahia et al., 2016), by temperature (Story et al., 2003; Kwan et al., 2006; Gracheva et al., 2010), and by GPCR second messenger signaling (Bandell et al., 2004; Wang et al., 2008; Wilson et al., 2011).

Overwhelming evidence from functional studies using selective ligands and knockouts, as well as single-cell transcript analyses, indicates that TRPA1 is expressed by a subset of sensory afferent neurons, including those in the dorsal root ganglion (DRG), vagal ganglion, and the trigeminal ganglion (Story et al., 2003; Jordt et al., 2004; Nassenstein et al., 2008; Usoskin et al., 2015; Kupari et al., 2019). TRPA1 agonists such as ROS (e.g., H_2_O_2_), electrophilic products of lipid peroxidation (e.g., 4-hydroyxynonenal) and electrophilic irritants [e.g., allyl isothiocyanate (AITC), cinnamaldehyde and acrolein; Bandell et al., 2004; Jordt et al., 2004; Bautista et al., 2006; Hinman et al., 2006; Andersson et al., 2008; Taylor-Clark et al., 2008a] cause TRPA1 activation in ∼15–50% of sensory neurons (depending on the ganglia/species), resulting in cation influx, depolarization, and the initiation of action potentials. Many if not all TRPA1-expressing afferents are small-diameter “nociceptive” neurons that also express the canonical capsaicin-sensitive ‘pain receptor’ TRP vanilloid 1 (V1), and thus the administration of TRPA1 agonists evokes pain and other nocifensive reflexes (Bautista et al., 2006; Kwan et al., 2006; McNamara et al., 2007; Bessac et al., 2008; Taylor-Clark et al., 2009; Liu et al., 2013). As such, TRPA1 plays an important role in the nocifensive responses of mammals to irritants, oxidative stress, and inflammation (Patapoutian et al., 2009; Wu et al., 2010), and has been a high-priority target for treating pain (Koivisto et al., 2022). Nevertheless, the precise characterization of TRPA1 expression in afferents and its relationship with other markers of nociceptive subsets [e.g., TRPV1, MrgprA3, MrgprD, substance P, and calcitonin gene-related peptide (CGRP)] has largely been based on studies of neurons following dissociation, which can alter transcript expression (Ono et al., 2012; Song et al., 2018). The characterization of TRPA1-expressing afferents *in vivo* has largely relied on TRPA1 antibodies that have low specificity.

Most initial screenings of TRPA1 transcript expression found little evidence in cell types other than sensory afferents (Story et al., 2003; Nagata et al., 2005; Jang et al., 2012). However, subsequent studies have demonstrated evidence of TRPA1 expression in multiple nonafferent cell types, including keratinocytes (Kwan et al., 2009), epithelium (Nassini et al., 2012), enterochromaffin cells (Cho et al., 2014; Bellono et al., 2017), cerebral artery endothelium (Earley et al., 2009), astrocytes (Shigetomi et al., 2012), Schwann cells (De Logu et al., 2017), and CNS neurons (Koch et al., 2011). In some cases, TRPA1 expression was identified using low-specificity antibodies or very low transcript numbers, and thus their determination is controversial. TRPA1 has been implicated in anxiety (de Moura et al., 2014), cognition, and memory (Lee et al., 2016a,b), and thus it is important to fully map its expression to take into account potential side effects of therapeutics developed to target TRPA1.

Here we have developed a *TRPA1^Flp^* reporter mouse, by replacing the endogenous TRPA1 stop codon with a 2A-Flp_O_ cassette. Flp_O_ is a codon modified and thermostable version of Flp recombinase (Raymond and Soriano, 2007). Similar to the commonly used Cre/lox system, Flp recombines specific FRT sites in DNA (Fenno et al., 2014), thus allowing for selective manipulation of targeted genes in TRPA1-expressing cells. By crossing the *TRPA1^Flp^* mouse with a reporter strain (*R26^ai65f^*) that expresses the fluorescent tdTomato in a Flp-sensitive manner, we have mapped TRPA1 expression to subsets of DRGs and vagal ganglia neurons innervating multiple peripheral organs, including the esophagus, gut, airways, bladder, and skin. Furthermore, chemogenetic activation of TRPA1-expressing fibers evokes acute nocifensive/pain behaviors. We found very limited tdTomato expression in other cell types, but most notably in subepithelial cells in the mucosa of the stomach and colon, although these did not express serotonin, a marker of enterochromaffin cells. Our *TRPA1^Flp^* mouse may prove useful for mapping and manipulating the contribution of TRPA1-expressing cells to physiological and pathophysiological processes.

## Materials and Methods

### Animals

**Figure 1.**
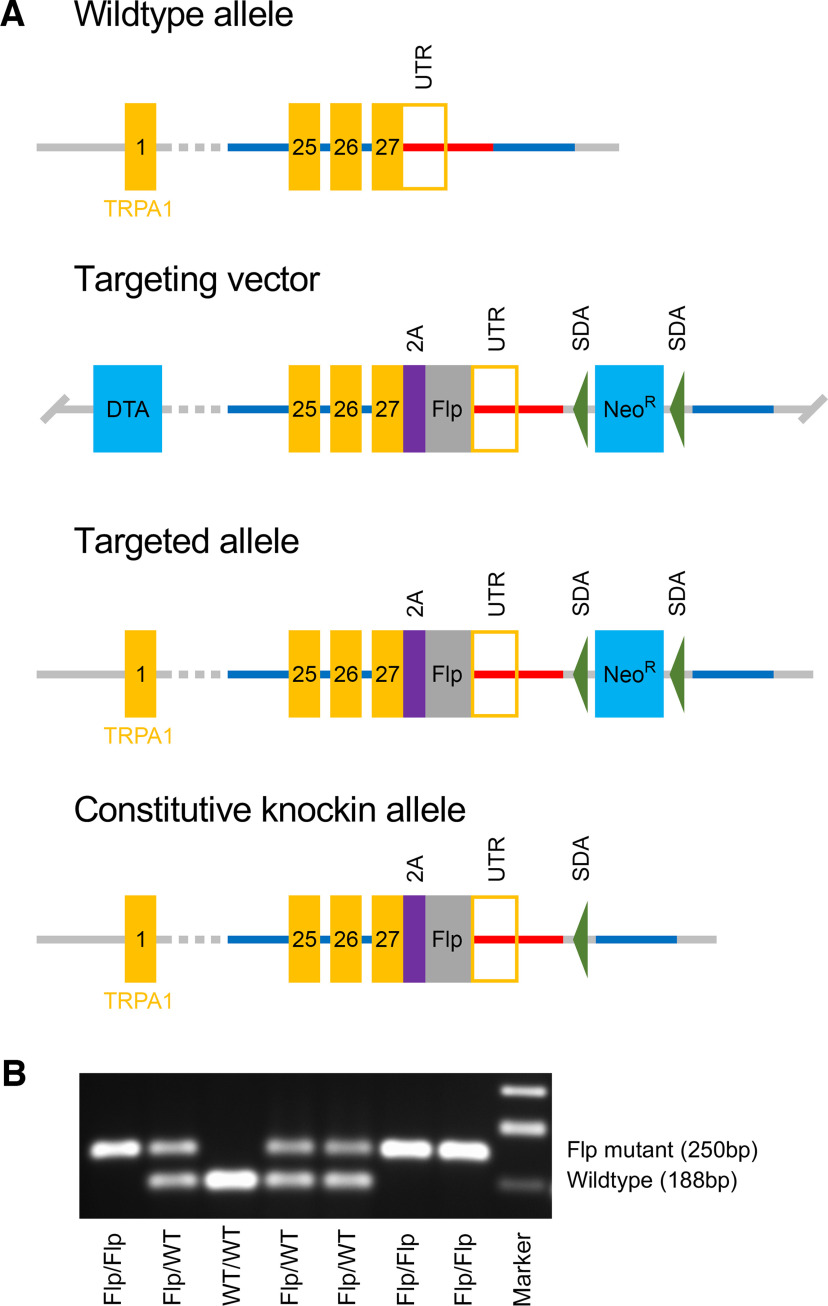
Development of the knock-in *TRPA1^Flp^* mouse. ***A***, Targeting strategy for the replacement of the TRPA1 TAG stop codon with a 2A-Flp cassette. Homology arms (blue and red lines) were generated for the TRPA1 gene [exons and 3′ untranslated region (UTR) in orange]. Homology arms of targeting vector include a neomycin resistance gene (Neo^R^) flanked by SDA sites for positive selection. A DTA fragment gene was placed in a nonhomologous region of targeting vector as a negative selection for nonhomologous recombination. ***B***, PCR of TRPA1 gene in offspring from a pairing of heterozygous *TRPA1^Flp^* mice. As expected, offspring have a Mendelian distribution of mutant (i.e., *TRPA1^Flp^*, at 250 bp) and wild-type (at 188 bp) alleles.

All procedures were in accordance with the animal protocols approved by the Institutional Animal Care and Use Committee. The gene for the murine TRPA1 receptor [National Center for Biotechnology Information (NCBI) Reference Sequence, NM_177781.5] is located on chromosome 1 and has 27 exons. To develop a knock-in mouse that expresses the codon-optimized Flp_O_ recombinase dependent on TRPA1 expression, the TAG stop codon in exon 27 was replaced with a 2A-Flp cassette (Fig. 1). The targeting vector homology arms were generated by high-fidelity Taq PCR using BAC (bacterial artificial chromosome) clones RP24-217G15 and RP24-312G12 from the C57BL/6J library as template. The targeting vector was assembled with recombination sites and selection markers: neomycin resistance gene (Neo^R^) flanked by self-deletion anchor (SDA) sites for positive selection and diphtheria toxin A (DTA) fragment gene for negative selection. Correct targeting vector synthesis was confirmed by appropriate digestion by restriction enzymes. The linearized vector was subsequently delivered to C57BL/6 ES cells via electroporation, followed by drug selection, PCR screening, and Southern blot confirmation. After gaining 184 neomycin-resistant clones, 12 potentially targeted clones were confirmed, 6 of which were expanded for Southern blotting. After confirming correctly targeted ES clones via Southern blotting, clones were selected for blastocyst microinjection, followed by founder production. Founders were confirmed as germline transmitted via crossbreeding with wild-type C57BL/6J mice. All aspects of knock-in mouse development were performed by Cyagen. Founders were mated to produce heterozygous and homozygous *TRPA1^tm1.1(flp)Ttc^* (MGI:7398201) mice (*TRPA1^Flp^*) in expected Mendelian proportions. These mice express TRPA1-2A-Flp_O_ from the endogenous TRPA1 gene. Upon translation, the 2A peptide self-cleaves (Furler et al., 2001) to release TRPA1 and Flp as separate peptides. *TRPA1^Flp^* mice develop normally and were observed to have no apparent phenotype. Homozygous *TRPA1^Flp^* mice were crossed with Flp-sensitive *R26^ai65f^* reporter mice [B6.Cg-*Gt(ROSA)26Sor^tm65.2(CAG-tdTomato)Hze^*/J; catalog #032864, The Jackson Laboratory; Daigle et al., 2018] to produce *TRPA1^Flp/+^R26^ai65f/+^* mice or with Flp-sensitive *R26^FLTG^* reporter mice [B6.Cg-*Gt(ROSA)26Sor^tm1.3(CAG-tdTomato,-EGFP)Pjen^*/J; catalog #026932, The Jackson Laboratory] to produce *TRPA1^Flp/+^R26^FLTG/+^* mice. Both the *R26^ai65f^* and *R26^FLTG^* mice have had an FRT-stop-FRT-tdTomato sequence knocked into the ROSA26 locus. Thus, Flp recombination evokes cell-specific expression of tdTomato. We also crossed the knock-in *TRPV1^Cre^* [B6.129 × 1-*Trpv1^tm1(cre)Bbm^/J*; catalog #017769, The Jackson Laboratory; Cavanaugh et al., 2011] with transgenic CAG-loxP-STOP-loxP-Gq-DREADD (designer receptors exclusively activated by designer drugs) mice [B6N;129-Tg(CAG-CHRM3*,-mCitrine)1Ute/J; catalog #026220, The Jackson Laboratory; formally known as Gt(ROSA)26Sor^tm2(CAG-CHRM3^*^,-mCitrine)Ute^/J] to produce TRPV1-hM3Dq mice that express the clozapine-*N*-oxide (CNO)-sensitive “activating” DREADD hM3Dq receptor in TRPV1-expressing cells. Last, knock-in *Pirt^Cre^* [*Pirt^tm3.1(cre)Xzd^*; a gift from Xinzhong Dong, Johns Hopkins University, Baltimore, MD; Kim et al., 2016] were crossed with the loxP-STOP-loxP-GCaMP6s mice [B6;129S6-*Gt(ROSA)26Sor^tm96(CAG-GCaMP6s)Hze^*/J; catalog #024106, The Jackson Laboratory) to produce *Pirt^Cre/+^R26^ai96/+^* mice, which express the cytosolic Ca^2+^ reporter GCaMP6s in all sensory neurons (Hadley et al., 2021). Specific alleles were confirmed by genotyping. Both male and female mice (6–10 weeks old) were used for all experiments, distributed equally. Offspring were weaned at 21 postnatal days, and up to four littermates were housed per cage under normal conditions (20°C, a 12 h dark/light cycle). Mice were provided with standard rodent chow and water *ad libitum*.

### Tissue collection and immunohistochemistry

Mice were killed by CO_2_ inhalation and transcardially perfused with ice-cold PBS. Vagal ganglia and DRGs from T1 to T5 and L3 to L5 were dissected out and fixed for 1 h in 3.7% formaldehyde (FA) at 4°C. Brainstem and spinal cord were dissected out and fixed for 4 h in 3.7% FA at 4°C. Brain, stomach, and colon were collected and fixed overnight in 3.7% FA at 4°C. Glabrous and hairy skin was removed from the hindpaw and fixed in 3.7% FA at 4°C overnight. For the cochlea, the temporal bones were collected and fixed in 4% PFA overnight at 4°C. The temporal bones were washed three times in PBS for 10 min and transferred to 10% EDTA, dissolved in H_2_O at pH 7.2, solution for overnight decalcification at 4°C. Tissue firmness was checked by pressing tissue with forceps to ensure adequate decalcification. The trachea, esophagus, and bladder were dissected out and cut longitudinally, with the ends pinned in a SYLGARD-covered dish and fixed in 3.7% FA overnight at 4°C. The muscle and the mucosal layers of the esophagus were carefully separated using fine scissors. The tissues were then unpinned and rinsed free floating three to four times at room temperature in PBS.

All tissues to be sliced using a cryostat were first cryoprotected in 20% sucrose. Stomach, colon, and skin were sectioned in 20 μm slices, ganglia were sectioned sagitally in 20 μm slices, brainstem and spinal cord were sectioned coronally in 40 μm slices, and brains were sectioned sagitally and coronally in 80 μm slices and collected onto Superfrost Plus slides. Slides were air dried at room temperature in the dark overnight. Slides were washed with PBS three times for 10 min, and tissue was permeabilized with 0.3% Triton X-100 in PBS (PBSTx) for 15 min followed by blocking for 1 h with 1% bovine serum albumin (BSA), 10% donkey serum (DS), and 0.3% PBSTx. Tissue was incubated with primary antibodies (Table 1) diluted in blocking buffer overnight at 4°C. After washing with 0.2% Tween 20 in PBS (PBST) three times for 10 min, the tissue was incubated with secondary antibodies (Table 1) in 1% BSA/5% DS in 0.2% PBST for 1 h. The tissue was washed with 0.2% PBST three times for 10 min and then, in some cases, counterstained with NeuroTrace fluorescent Nissl Stain for 1 h at 1:300 dilution in PBS. After washing with PBS, slides were air dried and mounted with DPX Mounting Medium (Sigma-Aldrich).

**Table 1:** Antibodies used in this study

Antibodies	Host	Dilution	Catalog no.	Source	RRID
Anti-DsRed	Rabbit	1:500	632496	Takara Bio	AB_10013483
Anti-RFP	Rabbit	1:1:200	600–401-379	Rockland	AB_2209751
Anti-GFP	Chicken	1:1000	ab13970	Abcam	AB_300798
Anti-GFP	Chicken	1:200	NB100-1614	Novus	AB_10001614
Anti-E-cadherin	Rat	1:300	ab11512	Abcam	AB_298118
Anti-α-smooth muscle actin	Goat	1:300	SAB2500963	MilliporeSigma	AB_10603763
Anti-CGRP	Rabbit	1:300	C8198	MilliporeSigma	AB_259091
Anti-5-HT	Goat	1:600	20079	ImmunoStar	AB_572262
Alexa Fluor 594 anti-CD11b	Rat	1:200	101254	BioLegend	AB_2563231
Alexa Fluor 488 anti-chicken Ig	Goat	1:500	A11039	Thermo Fisher Scientific	AB_2534096
Alexa Fluor 488 anti-rabbit Ig	Donkey	1:500	A21206	Thermo Fisher Scientific	AB_2535792
Alexa Fluor 546 anti-rabbit Ig	Donkey	1:500	A10040	Thermo Fisher Scientific	AB_2534016
Alexa Fluor 546 anti-rat Ig	Goat	1:500	A11081	Thermo Fisher Scientific	AB_141738
Alexa Fluor 647 anti-chicken Ig	Goat	1:500	Ab150171	Abcam	AB_2921318
Alexa Fluor 647 anti-rat Ig	Goat	1:500	A21247	Thermo Fisher Scientific	AB_141778
Alexa Fluor 647 anti-goat Ig	Chicken	1:500	A21469	Thermo Fisher Scientific	AB_2535872
Alexa Fluor 647 anti-rabbit Ig	Donkey	1:500	A31573	Thermo Fisher Scientific	AB_2536183
Biotin-XX conjugate anti-rabbit Ig	Goat	1:100	B2770	Molecular Probes	AB_2536431
Alexa Fluor 647 isolectin GS-IB4 (from *Griffonia simplicifolia*)	N/A	1:500	I32450	Thermo Fisher Scientific	SCR_014365
DyLight 594 Lycopersicon esculentum lectin (LE-lectin)	N/A	1:200	L32471	Thermo Fisher Scientific	AB_2336416
Neurotrace 435/455	N/A	1:300	N21479	Thermo Fisher Scientific	
Neurotrace 500/525	N/A	1:300	N21480	Thermo Fisher Scientific	

Complete lungs with attached bronchi and trachea were dissected out and fixed in 3.7% FA overnight at 4°C with gentle agitation. Lungs were washed with ice-cold PBS three times for 30 min, followed by cryoprotection in 30% sucrose solution. The lungs were flushed with PBS three times and inflated with 2% low melting agarose solution. After the agarose solution had solidified, the lung lobes were separated and snap frozen with OCT compound for cryosection. Eighty micrometer lung slices were collected in cryoprotectant-filled wells and stored in −20°C. Using identical blocking solutions, permeabilizing solutions, and antibody solutions (see above), lung slices were washed in PBS three times for 10 min and permeabilized for 20 min. Lung slices were then blocked for 1.5 h followed by primary antibody incubation overnight at 4°C. The slices were then washed three times for 20 min and incubated with secondary antibodies for 2 h. The slices were washed again and mounted onto glass slides with VECTASHIELD Antifade Mounting Medium with DAPI.

The staining of the esophageal muscle and mucosa, trachea and bladder were performed in 0.5 ml tubes. The washing in filtered PBS between staining steps was performed in 15 ml tubes on the rotor at room temperature. Tissues were permeabilized in 1% Tween 20 (Sigma-Aldrich) in filtered PBS at room temperature for 6 h and washed in filtered PBS (3 × 20 min on rotator). All tissues except trachea were then incubated in blocking solution [1% BSA in PBS (BSA/PBS)] with four drops of Avidin solution (Avidin/Biotin Blocking Kit; catalog #SP-2001, Vector Laboratories) and incubated at 4°C for 4 h. The tissues were then washed 5 × 20 min on rotator and then incubated with primary antibodies diluted in BSA/PBS with four drops of Biotin (as necessary) for 48 h at 4°C. The tissues were then washed in filtered PBS (10 × 30 min), incubated with secondary antibodies and/or goat biotin-XX conjugate anti-rabbit IgG (H + L) secondary antibody (catalog #B2770, Thermo Fisher Scientific) diluted 1:100 in BSA/PBS overnight at room temperature, then washed in PBS (10 × 20 min), incubated with streptavidin (Alexa Fluor 548 conjugate; catalog #S11223, Thermo Fisher Scientific) diluted 1:100 in filtered PBS for 5 h at room temperature; washed in filtered PBS (3 × 20 min); incubated in anti-fade, pH 8.6, glycerol (Sigma-Aldrich) for 24 h at room temperature; and stored at 4°C. The esophagus preparation was placed mucosal side up on a glass slide and covered with a coverslip (24 × 50 mm).

Tracheal tissue was blocked with BSA/PBS alone at 4°C for 4 h. The trachea was then washed 5 × 20 min on rotator and then incubated with primary antibodies diluted in BSA/PBS for 48 h at 4°C. The trachea was then washed in filtered PBS (10 × 30 min) and incubated with secondary antibodies in BSA/PBS overnight at room temperature, then washed in filtered PBS (3 × 20 min), incubated in anti-fade, pH 8.6, glycerol (Sigma-Aldrich) for 24 h at room temperature, and stored at 4°C.

In some cases, *TRPA1^Flp/+^R26^ai65f/+^* mice were administered an intraganglionic injection of the constitutively active AAV9-hSyn-GFP (∼2 × 10^13^ GC/ml; catalog #50465, Addgene) into the vagal ganglia to drive GFP expression in vagal neurons. Using a procedure that has been described previously (Kim et al., 2022), mice were anesthetized with ketamine (50 mg/kg) and dexmedetomidine (0.5 mg/kg) via intraperitoneal injection. An ∼2 cm incision was made over a shaved superficial portion of the masseter muscle area. The skin was retracted, and the vagus nerve was located and then separated from the common carotid artery and the anterior laryngeal nerve using a cotton tip. The virus microinjection assembly consisted of a pulled glass micropipette (tip diameter, ∼20 μm) attached to a 1 ml syringe via plastic tubing. The tip of the micropipette was gently inserted into the exposed vagal ganglia and then 0.7 μl of AAV9-hSyn-GFP was injected using ∼0.5 psi pressure. Three to 6 weeks later, the mice were used for analysis.

Images were taken with the Dragonfly Spinning Disk Confocal Microscope (Andor) equipped with a Zyla 4.2 PLUS sCMOS Camera (Andor; 2048 × 2048 pixel with 6.5 μm pixel size). The pinhole size was 25 μm. We used either a 10× UPLSAPO [0.4 numerical aperture (NA)], a 20× UPLSAPO (0.75 NA), or a 40× UPLSAPO (1.25 NA, silicone oil-immersion) objective was used, depending on the study. Fluorophores were excited by laser wavelengths at 405, 488, 561, or 637 nm. The *z*-stacked multitile images were stitched using either Fusion software or Imaris Stitcher. All 3D images were further processed using Imaris software. The identification of anatomic structures and subnuclei were based on the mouse brain map (Paxinos and Franklin, 2012). In mice, the nodose vagal ganglion and the jugular vagal ganglion are partially fused into a single structure. Nevertheless, these two ganglia can be approximately discriminated visually in sagittal slices of the vagal ganglia, as shown previously (Nassenstein et al., 2010; Kim et al., 2020a,b). Cell counts and somal diameter of neurons within the vagal ganglia and DRGs were obtained using Fiji software.

### Sensory ganglia dissociation

Mice were killed by CO_2_ asphyxiation followed by exsanguination. DRGs or vagal ganglia were isolated in Ca^2+^-free, Mg^2+^-free HBSS, incubated in HBSS containing type 1 collagenase (2 mg/ml) and dispase II (2 mg/ml), and then mechanically dissociated with fire-polished pipettes. Individual neurons were washed and resuspended in L-15 media supplemented with 10% fetal bovine serum (FBS), 100 U/ml penicillin, and 100 μg/ml streptomycin, then were plated onto poly-d-lysine-coated and laminin-coated coverslips. As a positive ganglionic control for the RT-PCR studies (see below), we transferred 2 μl of the cell suspension into PCR tubes containing 1 μl RNase OUT. Neurons were incubated at 37°C in antibiotic-free L-15 media supplemented with 10% FBS and used within 24 h.

### Single-neuron RT-PCR

Coverslips with dissociated vagal sensory neurons were superfused with Krebs bicarbonate solution (KBS; in mm: 118 NaCl, 5.4 KCl, 1 NaH_2_PO_4_, 1.2 MgSO_4_, 1.9 CaCl_2_, 25 NaHCO_3_, and 11 dextrose, gassed with 95% O_2_-5% CO_2_). tdTomato-expressing neurons were identified by fluorescence (554 nm excitation, 581 nm emission) and a BX51WI Microscope (Olympus). Individual neurons were collected with a borosilicate glass pipette by applying a gentle negative pressure. Each single neuron was stored in a PCR tube containing 1 μl RNase OUT and stored at −80°C. The KBS surrounding the vagal neurons was sampled (∼2 μl) as a negative control for the RT-PCR.

First-strand cDNA was synthesized with SuperScript III First-Strand Synthesis System for RT-PCR by following the manufacturer instructions. Primers and dNTP (deoxynucleotide triphosphate) mix were added into each neuron-containing PCR tube. Samples were incubated at 75°C for 10 min and then placed on ice for at least 1 min. cDNA synthesis mix containing 10× reverse transcriptase (RT) buffer, MgCl_2_, DTT, and SuperScript III RT was added into each sample. Samples were incubated at 50°C for 50 min, followed by 85°C for 5 min. cDNA was stored at −20°C until used for PCR amplification. cDNA was amplified with HotStarTaq DNA polymerase for 50 cycles of denaturation at 94°C for 30 s, followed by annealing at 60°C for 30 s and extension at 72°C for 1 min. Customized intron-spanning primers for mouse TRPA1, TRPV1, P2X_2_, preprotachykinin A (PPT-A), and vesicular glutamate transporter 2 (VGluT2; Table 2) were used as previously reported (Nassenstein et al., 2008, 2010). Products were visualized in 1.5% agarose gel with GelRed, with a 100 bp marker. VGluT2 was used as a positive control.

**Table 2:** Primers used for single-cell RT-PCR in mouse neurons

Gene	Primer	Sequence 3′ to 5′	Product size	NCBI reference sequence
TRPA1	Forward	GGAGCAGACATCAACAGCAC	393 bp	AY231177
	Reverse	GCAGGGGCGACTTCTTATC		
TRPV1	Forward	TCACCGTCAGCTCTGTTGTC	229 bp	NM 001001445
	Reverse	GGGTCTTTGAACTCGCTGTC		
P2X2	Forward	GGGGCAGTGTAGTCAGCATC	241 bp	NM_153400
	Reverse	TCAGAAGTCCCATCCTCCA		
PPT-A	Forward	AGACCCAAGCCTCAGCAGTT	215/171/162/118 bp	D 17584
	Reverse	CGTCTTCTTTCGTAGTTCTGCATT		
VGluT2	Forward	CTGCGATACTGCTCACCTCTAC	175 bp	AF324864
	Reverse	GCCAACCTACTCCTCTCCAA		

### FURA-2AM Ca^2+^ imaging and analysis

Neuron-coated coverslips were incubated with 4 μm fura-2 AM for 30–60 min at 37°C. Coverslips were loaded into a chamber on an inverted microscope (model Eclipse Ti, Nikon), fitted with a CFI Super Fluor 10× objective and a CoolSnap HQ2 camera (Photometrics), and perfused with heated (33–34°C) HEPES buffer (154 mm NaCl, 1.2 mm KCl, 1.2 mm MgCl_2_, 2.5 mm CaCl_2_, 5.6 mm d-glucose). Cells were subjected to fluorescent light delivered by a 300 W (5000 lumens) PE300BFA Xenon Parabolic Bulb housed in a Lambda LS (Sutter Instruments), with appropriate excitation and emission filters. Bright-field and fluorescence images (535 nm excitation, 610 nm emission) were collected to facilitate subsequent analysis and the determination of tdTomato expression, respectively. Changes in [Ca^2+^]_i_ were monitored every 6 s using sequential excitation at 340 and 380 nm (510 nm emission) and evaluated ratiometrically using the 340:380 ratio (R). All drugs were diluted in HEPES buffer. AITC (100 μm) was used to determine the functional expression of TRPA1 (Jordt et al., 2004), and capsaicin (1 μm) was used to determine the functional expression of TRPV1 (Caterina et al., 1997). Neurons were further characterized by response to KCl (75 mm) before ionomycin (5 μm), which evoked a maximal Ca^2+^ response. Region of interest (ROI) analysis for individual neurons was performed using Nikon Elements. ROIs with an unstable, high, or noisy baseline were eliminated from analysis. Neurons that failed to exhibit an increase in [Ca^2+^]_i_ to AITC, capsaicin, or KCl challenges (>30% the ionomycin maximal response) were eliminated. An individual neuron was considered to be sensitive to a given agent if *R*_agent_ > (*R*_bl_ + 2 * SD_bl_) + 0.03; where *R*_agent_ is the average 340:380 ratio during treatment, *R*_bl_ is the average 340:380 ratio before treatment, and SD_bl_ is the SD of R_bl_. Neurons were grouped by tdTomato expression and sensitivity to AITC and capsaicin. In some cases, the “δ” evoked response to a given treatment was calculated as the *R*_agent_ – *R*_bl_.

### GCaMP6s Ca^2+^ imaging and analysis

Ca^2+^ transients in individual neuronal soma in intact ganglia were assessed using the Ca^2+^-sensitive fluorescent protein GCaMP6s. In some experiments, *Pirt^Cre/+^R26^ai96/+^* mice were used to drive GCaMP6s expression in all sensory neurons. In other experiments, GCaMP6s expression was induced in vagal neurons of *TRPA1^Flp/+^R26^ai65f/+^* mice by intraganglionic injection with the constitutively active AAV9-CAG-GCaMP6s (∼2 × 10^13^ GC/ml; catalog #100844, Addgene), using the technique described above.

Mice were killed by CO_2_ inhalation and exsanguination, and the vagal ganglia and T2, T3, or L5 DRGs were dissected out and pinned in a chamber superfused with KBS (4 ml/min at 37°C). The ganglion was visualized using a 25× water-immersion objective and the Olympus FVMPE-RS multiphoton system. The tissue was excited with 960nm (InSight X3 IR laser), and the emitted light was split into two paths using a beam splitter, which was then detected with specific filter cubes and distinct photomultiplier tubes (PMT) containing gallium arsenide phosphide (GaAsP): an FVG filter cube and 488 PMTs for GCaMP6s, and an FGR filter cube and 555 PMTs for tdTomato. A *z*-stack (100 μm) of the ganglia was visualized using Fluoview software (Olympus), which allowed for automated increases in laser percentage and PMT voltage depending on the depth of the *z*-plane into the ganglia. Live images were recorded every 12 s. For the vagal recordings of *TRPA1^Flp/+^R26^ai65f/+^* mice with intraganglionic injection of AAV9-CAG-GCaMP6s, we determined the GCaMP6s responses to AITC (100 μm, 300 s) and then KCl (50 mm, 300 s) added to the ganglionic compartment. For the DRG recordings of *Pirt^Cre/+^R26^ai96/+^* mice, we determined the GCaMP6s responses to AITC (100 μm, 300 s), followed by capsaicin (1 μm, 300 s) and then KCl (50 mm, 300 s) added to the ganglionic compartment.

Fluoview-derived images were analyzed offline using Fiji software (ImageJ). Each ganglion was imaged up to a depth of 100 μm, in ten 10-μm-thick *z*-planes. We analyzed either odd-numbered or even-numbered *z*-planes. This limited the possibility that a given mouse neuron (which are ∼20 μm in diameter) was counted twice. Each analyzed *z*-plane was used to mark the ROIs of tdT^+^ neurons and neurons that responded to any of the treatments. A neuron was considered to be sensitive to a given treatment if its maximal GCaMP6s response during treatment was double the mean baseline GCaMP6s fluorescence of all identified neurons within that ganglion. A neuron was only included in the analysis if it responded to one of the treatments. For each neuron, the mean baseline GCaMP6s fluorescence was subtracted from the GCaMP6s fluorescence at each time point for dataset comparisons (F_1_-F_0_).

### Nocifensive/pain behavioral assessment

The nocifensive behavior of mice following intraplantar injection of the hindpaw with vehicle or CNO dihydrochloride (catalog #6329, Tocris Bioscience) was determined. In some experiments, *TRPA1^Flp/Flp^* mice first received an intraplantar injection (10 μl) into the right hindpaw with rAAV2-hSyn-fDIO-hM3Dq [∼1 × 10^13^ genome copies (GC)/ml; catalog #154868, Addgene] or (as a control) rAAV2-Ef1a-fDIO-mCherry (∼1 × 10^13^ GC/ml; catalog #114471, Addgene) 4 weeks before the behavioral assessment to drive the Flp-dependent expression of the “activating” DREADD hM3Dq receptor (or mCherry as control) in TRPA1-expressing paw afferents. CNO working solution was made fresh on the day of the experiment in sterile PBS (1 or 10 μg). Mice received an intraplantar injection (20 μl) of CNO (1 or 10 μg) or vehicle (sterile PBS) into the right hindpaw and then were placed immediately into a clear cage monitored by a camera placed underneath. The duration (in seconds) that the animal spent flinching the injected hindpaw over a 15 min period was recorded by the camera. The recordings were then scored by an observer blinded to treatment allocation and mouse strain. For each experiment, the response to vehicle and CNO was recorded in the same set of mice, with the treatments separated by 1 week (paired experiments). Thus, the following studies were performed: (1) TRPV1-hM3Dq mice (*n* = 5: two females, three males) given intraplantar injection of vehicle and 1 μg of CNO (paired experiments); (2) *TRPA1^Flp/Flp^* mice 4 weeks after intraplantar injection with rAAV2-Ef1a-fDIO-mCherry (*n* = 3: one female, two males) given intraplantar injection of vehicle and 1 μg of CNO (paired experiments); and (3) *TRPA1^Flp/Flp^* mice 4 weeks after intraplantar injection with rAAV2-hSyn-fDIO-hM3Dq (*n* = 6: four females, two males) given intraplantar injection of vehicle and 1 μg of CNO and 10 μg of CNO (paired experiments).

### Statistical analysis

All data were analyzed in GraphPad Prism 10. The somal areas of various vagal and DRG afferent subpopulations were compared using ANOVA with Tukey’s multiple-comparisons test. In the fura-2 AM studies, the mean δ responses to a given irritant were compared between tdT^+^ neurons and tdT^–^ neurons using an unpaired *t* test. The time spent flinching after intraplantar injection of CNO was compared against vehicle responses in paired experiments: either *t* test for single CNO doses or ANOVA with Dunnett’s multiple-comparisons test for multiple CNO dose studies. In all cases, a *p* value <0.05 was considered significant.

## Results

### tdTomato expression in sensory ganglia of TRPA1 reporter mice

**Figure 2.**
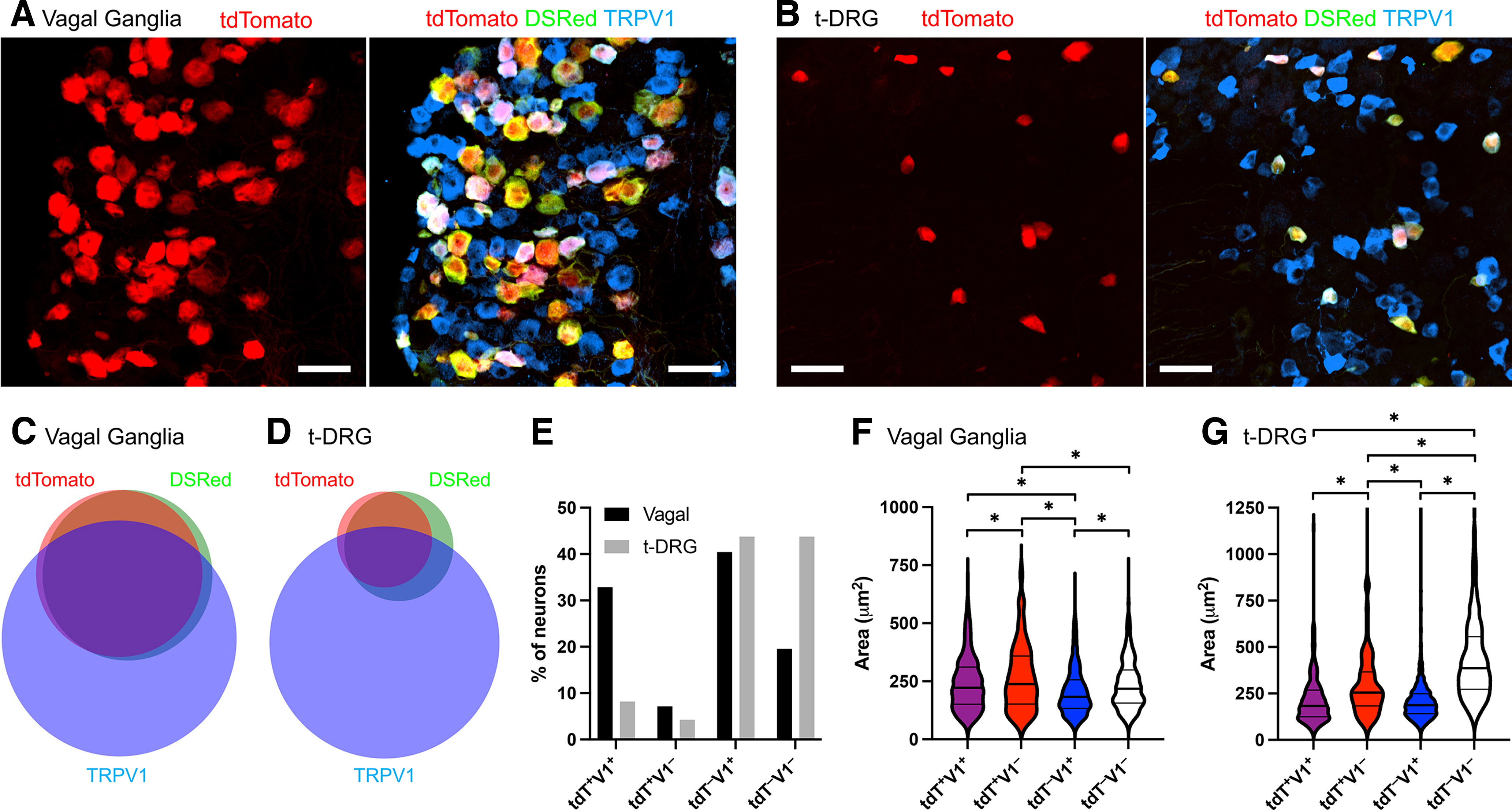
Subsets of sensory neurons in *TRPA1^Flp^R26^ai65f^* mice express tdTomato. ***A***, ***B***, Representative slices of native tdTomato expression (in red) compared with antibodies against tdTomato (DsRed; green) and TRPV1 (blue) in vagal ganglia (***A***) and thoracic DRGs (***B***). Scale bars, 50 μm. ***C***, ***D***, Euler diagram showing the overlap of neurons expressing native tdTomato and immunoreactivity for tdTomato (DsRed) and TRPV1 in vagal ganglia (***C***) and thoracic DRGs (***D***). ***E***, Percentage of vagal and DRG neurons (*n* = 1442 and *n* = 2635, respectively) that express tdTomato and/or TRPV1. ***F***, ***G***, Violin plots of the cell body area (in square micrometers) of neurons expressing tdTomato and/or TRPV1 in the vagal ganglia (***F***) and thoracic DRGs (***G***). *Significant difference in cell body area (*p* < 0.05).

To investigate the expression of TRPA1, we generated a knock-in *TRPA1^Flp^* mouse (Fig. 1), which was crossed with the Flp-sensitive *R26^ai65f^* reporter mouse to make *TRPA1^Flp/+^R26^ai65f/+^* mice, which express the red fluorescent tdTomato in TRPA1-expressing cells. Serial sections of sensory vagal ganglia (7 slices from three mice) and thoracic DRGs (t-DRGs; 12 slices from three mice) demonstrated native tdTomato expression in a subset of neurons (Fig. 2*A*,*B*). In general, the tdTomato signal-to-noise ratio in sensory ganglia was high: comparing antibody detection (anti-DsRed) with the native tdTomato showed that 91.9% (530 of 577 neurons) of vagal and 71.1% (234 of 329) of t-DRG tdTomato expression was detectable from the native fluorescence alone (Fig. 2*C*,*D*). In total, 40.0% (577 of 1442) of vagal and 12.5% (329 of 2635) of t-DRG neurons expressed tdTomato. Counterstaining sensory ganglia with an antibody against TRPV1 showed that 44.8% (474 of 1057) of TRPV1^+^ vagal neurons (Fig. 2*C*,*E*) and 15.8% (216 of 1369) of TRPV1^+^ thoracic t-DRG neurons also expressed tdTomato (Fig. 2*D*,*E*). Nevertheless, a noteworthy percentage of TRPA1^Flp^-tdTomato^+^ neurons in vagal ganglia (17.9%, 103 of 577) and t-DRGs (34.3%, 113 of 329) were TRPV1^–^. Although there was substantial variance in the size of vagal neurons in the four groups categorized by TRPA1^Flp^-tdTomato and TRPV1 expression, there were significant differences between the groups, as follows: tdT^+^TRPV1^–^ > tdT^+^TRPV1^+^ = tdT^–^TRPV1^–^ > tdT^–^TRPV1^+^ (*p* < 0.05; Fig. 2*F*), indicating that vagal TRPA1^Flp^-tdT^+^ neurons were larger than their TRPA1^Flp^-tdT^–^ counterparts, regardless of TRPV1 expression. Size relationships were different in t-DRG neurons: tdT^–^TRPV1^–^ ≫ tdT^+^TRPV1^–^ > tdT^+^TRPV1^+^ = tdT^–^TRPV1^+^ (*p* < 0.05; Fig. 2*G*). Native tdTomato expression was also observed in 7.9% (393 of 4996) of lumbar DRG (l-DRG) neurons (data not shown).

**Figure 3.**
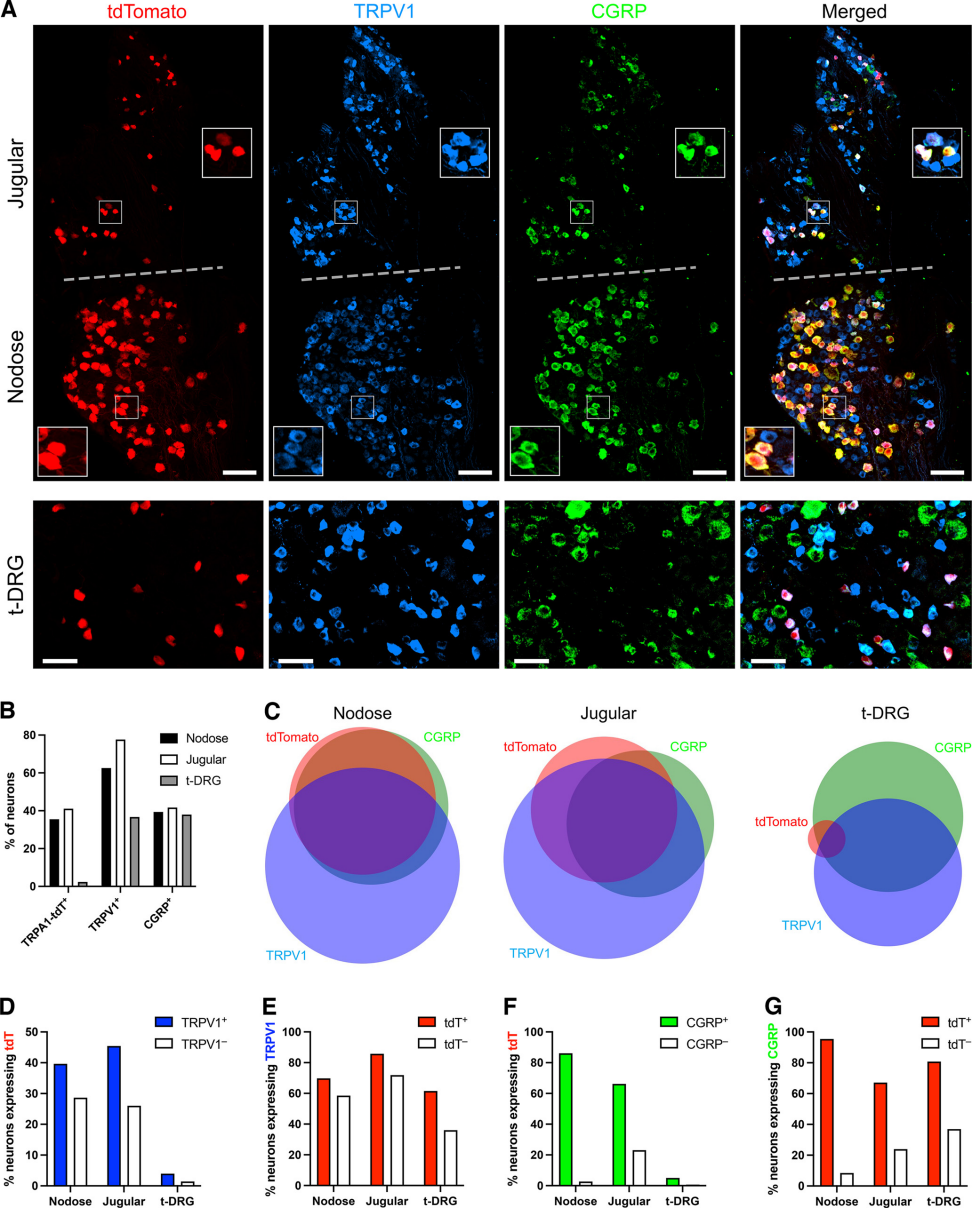
Overlap of TRPV1 and CGRP with tdTomato in sensory neurons from *TRPA1^Flp^R26^ai65f^* mice. ***A***, Representative slices of tdTomato (red), TRPV1 (blue), and CGRP (green) expression in vagal ganglia (top) and thoracic DRGs (bottom). Scale bars: top, 100 μm; bottom, 50 μm. The vagal ganglion is visually divided by a dotted gray line into the nodose and jugular portions, each with a higher magnification insert. ***B***, Percentage of nodose, jugular, and DRG neurons (*n* = 988, *n* = 326, and *n* = 1093, respectively) that express tdTomato, TRPV1, or CGRP. ***C***, Euler diagram showing the overlap of neurons expressing tdTomato, TRPV1, and CGRP in nodose ganglia, jugular ganglia, or DRGs. ***D–G***, Percentage of neurons in the nodose ganglia, jugular ganglia, or DRGs sharing expression of two markers: tdTomato expression in TRPV1^+^ and TRPV1^–^ neurons (***D***); TRPV1 expression in tdT^+^ and tdT^–^ neurons (***E***); tdTomato expression in CGRP^+^ and CGRP^–^ neurons (***F***); and CGRP expression in tdT^+^ and tdT^–^ neurons (***G***).

We further compared the expression of tdTomato in the sensory neurons of *TRPA1^Flp/+^R26^ai65f/+^* mice with the expression of the neuropeptide CGRP and TRPV1 (Fig. 3*A–C*; six DRG slices and four vagal slices from three mice). The vagal ganglion is composed of the anatomically distinct nodose ganglion and the jugular ganglion. tdTomato was expressed in 245 of 618 (39.6%) TRPV1^+^ nodose neurons and in 106 of 370 (28.6%) TRPV1^–^ nodose neurons (Fig. 3*C*,*D*), whereas tdTomato was expressed in 115 of 253 (45.5%) TRPV1^+^ jugular neurons and in 19 of 73 (26.0%) TRPV1^–^ jugular neurons, indicating that the percentage of TRPA1^Flp^-tdT^+^ neurons expressing TRPV1 was greater in jugular neurons (85.8%) compared with nodose neurons (69.8%; Fig. 3*C*,*D*). TRPV1 expression was only slightly more prevalent in tdT^+^ than tdT^–^ neurons in the nodose and jugular ganglia (Fig. 3*E*). We noted that tdTomato expression in the nodose ganglion was almost perfectly correlated with CGRP expression (Fig. 3*C*,*F*,*G*), as follows: CGRP was expressed by 335 of 351 (95.4%) tdT^+^ nodose neurons and tdTomato was expressed by 335 of 389 (86.1%) CGRP^+^ nodose neurons. Although overall CGRP expression was similar between nodose and jugular ganglia (Fig. 3*B*), this association was less robust in the jugular ganglion, as follows: CGRP was expressed by 90 of 134 (67.2%) tdT^+^ jugular neurons and tdTomato was expressed by 90 of 136 (66.2%) CGRP^+^ jugular neurons. Similar to the previous data, TRPA1^Flp^-tdTomato expression was limited in t-DRGs (Fig. 3*A*,*B*), as follows: tdTomato was expressed in only 16 of 401 (4.0%) TRPV1^+^ t-DRG neurons and in 10 of 692 (1.4%) TRPV1^–^ t-DRG neurons (Fig. 3*C*,*D*). Nevertheless, there was a positive association of tdTomato and CGRP expression, as follows: CGRP was expressed by 21 of 26 (80.8%) tdT^+^ t-DRG neurons (Fig. 3*C*,*G*). Indeed, the association of tdTomato expression in t-DRG neurons was stronger with CGRP than with TRPV1, as follows: 15 of 16 (93.8%) tdT^+^TRPV1^+^ t-DRG neurons expressed CGRP and 6 of 10 (60.0%) tdT^+^TRPV1^–^ t-DRG neurons expressed CGRP.

### Correlation of TRPA1 expression and tdTomato expression in sensory ganglia of TRPA1 reporter mice

**Figure 4.**
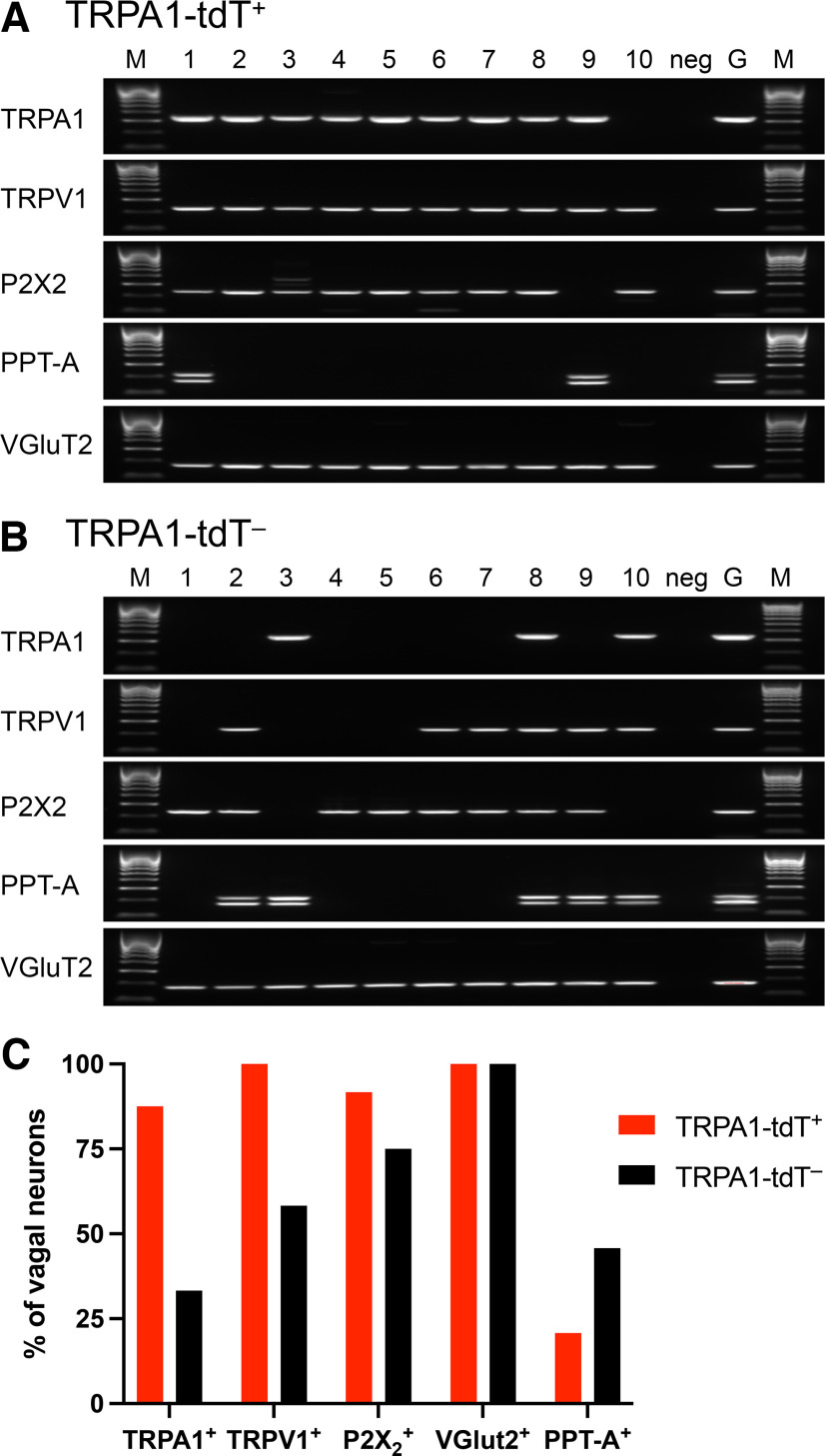
Individual tdT^+^ vagal neurons from *TRPA1^Flp^R26^ai65f^* mice express TRPA1. ***A***, ***B***, Representative gels of individual dissociated vagal neurons (1–10 tdT^+^, ***A***; 1–10 tdT^–^, ***B***) following RT-PCR for transcripts for TRPA1 (393 bp), TRPV1 (229 bp), P2X2 (241 bp), PPT-A (215/171/162/118 bp), and VGluT2 (175 bp). Also shown are Hyperladder 100 bp (M), KBS negative control (neg), and whole ganglia positive control (G). ***C***, Percentage of tdT^+^ and tdT^–^ vagal neurons (*n* = 24 each) that express TRPA1, TRPV1, P2X2, PPT-A, and VGluT2.

**Figure 5.**
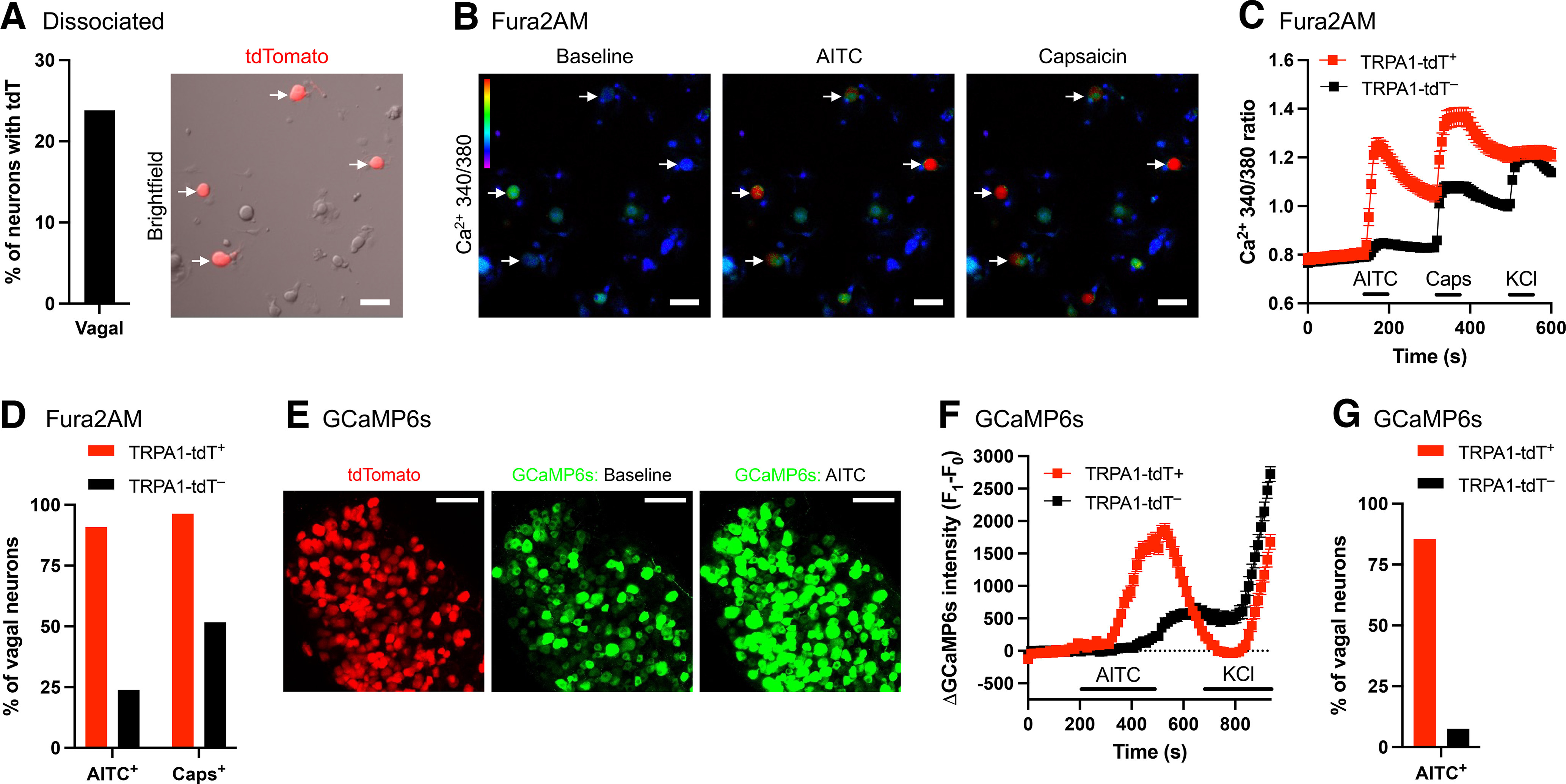
tdTomato expression correlates with sensitivity to AITC in vagal neurons from *TRPA1^Flp^R26^ai65f^* mice. ***A***, Percentage of dissociated vagal neurons (*n* = 231) expressing tdTomato, and representative bright-field image of neurons with overlaid tdTomato fluorescence. Scale bar, 50 μm. Arrows, tdT^+^ neuron. ***B***, Representative ratiometric 340:380 [Ca^2+^]_i_ images for the neurons shown in ***A*** under baseline conditions, and following treatment with AITC (100 μm) and capsaicin (1 μm). ***C***, Mean ± SEM 340:380 ratio [Ca^2+^]_i_ responses of dissociated tdT^+^ (red; *n* = 55) and tdT^–^ (black; *n* = 176) vagal neurons to AITC (100 μm), capsaicin (Caps; 1 μm), and KCl (75 mm). ***D***, Percentage of dissociated tdT^+^ and tdT^–^ vagal neurons that were activated by AITC and capsaicin. ***E***, Representative *z*-stack images of intact vagal ganglia of *TRPA1^Flp^R26^ai65f^* mice with intraganglionic injection of AAV9-CAG-GCaMP6s showing tdTomato expression and the GCaMP6s fluorescence during baseline conditions and following treatment with AITC (100 μm). Scale bar, 100 μm. ***F***, Mean ± SEM GCaMP6s [Ca^2+^]_i_ responses of tdT^+^ (red; *n* = 144) and tdT^–^ (black; *n* = 66) neurons within intact vagal ganglia to AITC (100 μm) and KCl (50 mm). ***G***, Percentage of tdT^+^ and tdT^–^ neurons within intact vagal ganglia that were activated by AITC.

We sought to correlate tdTomato expression in the sensory ganglia of *TRPA1^Flp/+^R26^ai65f/+^* mice with evidence of TRPA1 expression. We first performed single-cell RT-PCR on 48 dissociated vagal neurons (from six mice), all of which expressed the VGluT2 as a positive control (Fig. 4). Twenty-one of the 24 (87.5%) tdT^+^ neurons expressed TRPA1, whereas 8 of the 24 (33.3%) tdT^–^ neurons expressed TRPA1. As such, 21 of the 29 (72.4%) TRPA1-expressing neurons were tdT^+^. TRPV1 expression was also higher in the tdT^+^ population (100%) compared with the tdT^–^ population (58.3%). Based on widespread expression of P2X_2_ and the limited expression of Tac1, it is likely that most of these vagal neurons were derived from the nodose ganglia (Taylor-Clark, 2021). We then performed fura-2 AM calcium imaging of dissociated vagal neurons (from three mice) in response to the TRPA1 agonist AITC (100 μm) and the TRPV1 agonist capsaicin (1 μm). Fifty-five of 231 (23.8%) dissociated vagal neurons expressed tdTomato (Fig. 5*A*). AITC evoked a robust increase in [Ca^2+^]_cyt_ in the TRPA1^Flp^-tdT^+^population compared with tdT^–^ neurons (Fig. 5*B*,*C*). Analysis of individual responses showed that AITC activated 39.8% (92 of 231) of vagal neurons in total. Importantly, 90.9% (50 of 55) of TRPA1^Flp^-tdT^+^ vagal neurons were activated by AITC compared with only 23.9% (42 of 134) of tdT^–^ neurons (Fig. 5*D*). As such, 50 of the 92 (54.3%) AITC-sensitive neurons were tdT^+^. When we compared the magnitude of AITC-evoked responses in neurons defined as AITC sensitive, we found that tdT^+^ neurons had greater mean δ responses (0.384 ± 0.036, *n* = 50) compared with tdT^–^ neurons (0.139 ± 0.014, *n* = 42, *p* < 0.05). Many tdT^+^ vagal neurons (96.4%, 53 of 55) were also activated by capsaicin, as were 51.7% (94 of 176) of the tdT^–^ population.

These data suggest that although tdT^+^ neurons in dissociated cultures are highly likely (∼90%) to express TRPA1 or be AITC-sensitive, a significant percentage of tdT^–^ neurons (∼30%) also express TRPA1 or are AITC sensitive. TRPA1 and tdT expression in dissociated neuronal soma may not precisely reflect native expression. To investigate native TRPA1 functionality, we studied vagal neuron Ca^2+^ fluxes reported by GCaMP6s in the intact ganglion, whose expression was induced by vagal ganglionic injection of *TRPA1^Flp/+^R26^ai65f/+^* mice with AAV9-CGaMP6s. AITC (100 μm) was added directly to the vagal ganglia *ex vivo*. In total, we recorded GCaMP6s fluorescence in 210 vagal neurons (from four mice), 144 of which expressed tdTomato (68.6%; Fig. 5*E*), suggesting somewhat preferential transfection by the AAV9. AITC evoked Ca^2+^ influx in 123 of 144 (85.4%) tdT^+^ neurons and in 5 of 66 (7.6%) tdT^–^ neurons (Fig. 5*F*,*G*). As such 123 of 128 (96.1%) AITC-sensitive neurons were tdT^+^. These vagal GCaMP6s experiments indicate that <10% of vagal tdT^–^ neurons express TRPA1 *in situ*.

**Figure 6.**
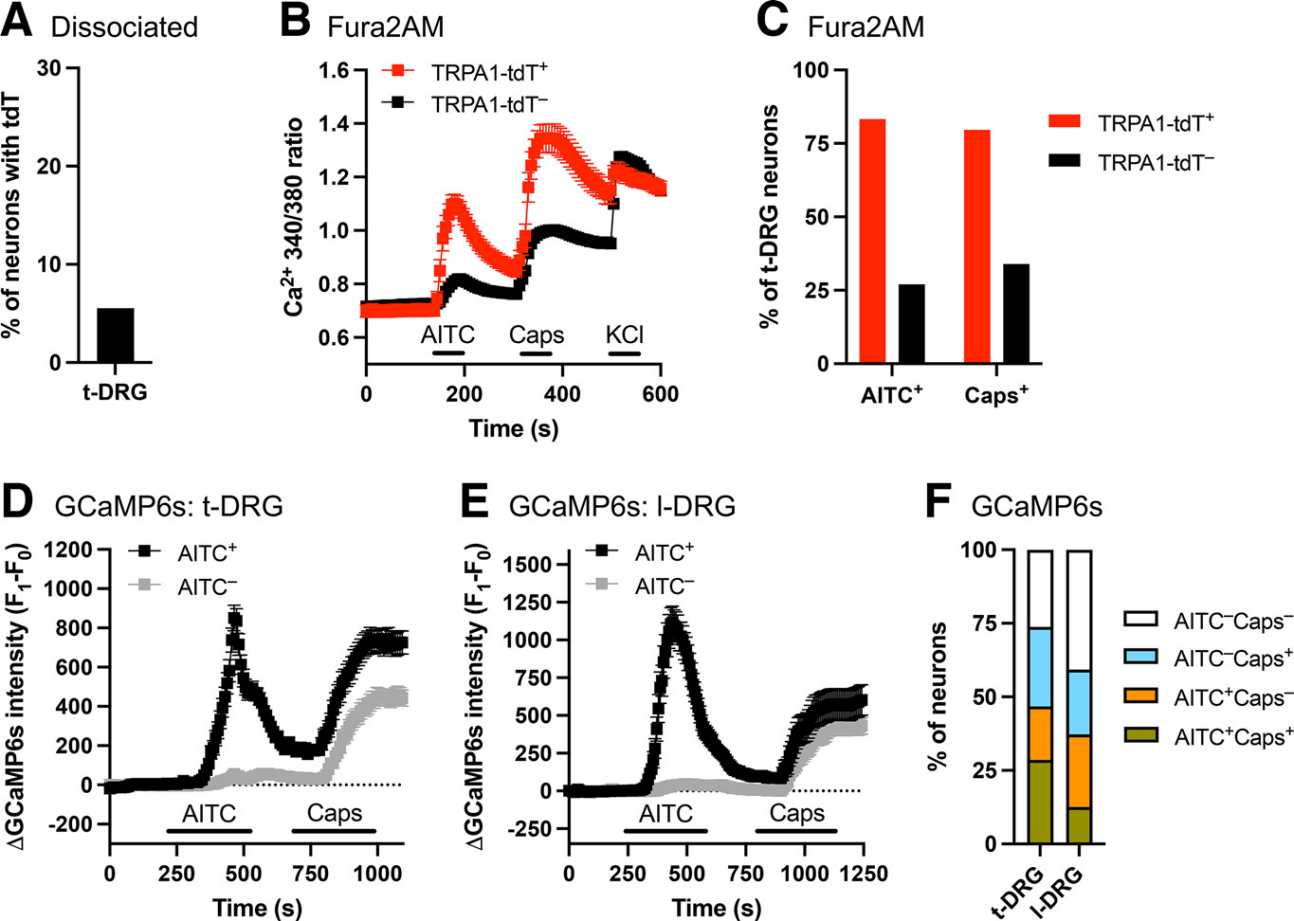
tdTomato expression correlates with sensitivity to AITC in DRG neurons from *TRPA1^Flp^R26^ai65f^* mice. ***A***, Percentage of dissociated thoracic DRG neurons (*n* = 976) expressing tdTomato. ***B***, Mean ± SEM 340:380 ratio [Ca^2+^]_i_ responses of dissociated tdT^+^ (red; *n* = 54) and tdT^–^ (black; *n* = 922) DRG neurons to AITC (100 μm), capsaicin (Caps; 1 μm), and KCl (75 mm). ***C***, Percentage of dissociated tdT^+^ and tdT^–^ DRG neurons that were activated by AITC and capsaicin. ***D***, Mean ± SEM GCaMP6s [Ca^2+^]_i_ responses of AITC-sensitive (black; *n* = 216) and AITC-insensitive (gray; *n* = 246) neurons within intact thoracic DRGs of *Pirt^Cre/+^R26^ai96/+^* mice to AITC (100 μm) and capsaicin (1 μm). ***E***, Mean ± SEM GCaMP6s [Ca^2+^]_i_ responses of AITC-sensitive (black; *n* = 86) and AITC-insensitive (gray; *n* = 145) neurons within intact lumbar DRGs of *Pirt^Cre/+^R26^ai96/+^* mice to AITC (100 μm) and capsaicin (1 μm). ***F***, Percentage of neurons within intact t-DRGs and l-DRGs that were activated by AITC and capsaicin.

We then performed fura-2 AM calcium imaging of dissociated t-DRG neurons (from nine mice) in response to the AITC (100 μm) and capsaicin (1 μm). Consistent with the immunohistochemistry data of t-DRG slices, only 5.5% (54 of 976) of dissociated t-DRG neurons expressed tdTomato (Fig. 6*A*). In total, AITC evoked calcium responses in 31.9% (294 of 976) DRG neurons. Importantly, AITC evoked greater responses in the TRPA1^Flp^-tdT^+^ population compared with tdT^–^ neurons: 83.3% (45 of 54) of TRPA1^Flp^-tdT^+^ t-DRG neurons were activated by AITC compared with only 27.0% (249 of 922) of tdT^–^ neurons (Fig. 6*B*,*C*). Nevertheless, only 45 of the 294 (15.3%) AITC-sensitive neurons were tdT^+^. We compared the magnitude of AITC-evoked responses in neurons defined as AITC-sensitive: tdT^+^ neurons had greater mean δ responses (0.365 ± 0.035, *n* = 45) compared with tdT^–^ neurons (0.195 ± 0.008, *n* = 249, *p* < 0.05). Again, many tdT^+^ neurons (79.6%, 43 of 54) and some of the tdT^–^ neurons (33.9%, 313 of 922) from the t-DRG were capsaicin sensitive.

Given the mismatch between tdTomato expression and TRPA1 functionality in dissociated DRG neurons, we also assessed TRPA1 expression by recording DRG neuron Ca^2+^ fluxes reported by GCaMP6s in the intact ganglion. Because of technical issues with injecting AAV (adeno-associated virus) into the thoracic and lumbar ganglia, we chose instead to assess the percentage of neurons with AITC sensitivity in the intact DRGs of *Pirt^Cre/+^R26^ai96/+^* mice (*n* = 5 animals), which express CGaMP6s in >95% of sensory neurons. AITC (100 μm) was added directly to the DRGs *ex vivo*. AITC evoked Ca^2+^ influx in 216 of 462 (46.8%) t-DRG neurons (Fig. 6*D*,*F*). Similarly, AITC evoked Ca^2+^ influx in 86 of 231 (37.2%) l-DRG neurons (Fig. 6*E*,*F*). Thus, the percentage of AITC-responsive DRG neurons in intact ganglia is similar to acutely dissociated DRG cultures. Therefore, the observation that the percentage of AITC-sensitive DRG neurons (32–47%) is much greater than the percentage of tdT^+^ neurons in the DRGs of *TRPA1^Flp/+^R26^ai65f/+^* mice (5–12%) is likely because of a lack of efficiency in the tdT expression in the DRGs of *TRPA1^Flp/+^R26^ai65f/+^* mice.

Last, we crossed the *TRPA1^Flp^* mouse with the Flp-sensitive *R26^FLTG^* reporter mice to produce *TRPA1^Flp/+^R26^FLTG/+^* mice. Serial sections of the t-DRGs from these mice (six slices from three mice) demonstrated that 4.4% (61 of 1385) of neurons expressed tdTomato (data not shown), which is similar to data from *TRPA1^Flp/+^R26^ai65f/+^* mice. This indicates that the lack of efficiency in the tdT expression in DRG neurons is not dependent on the specific Flp-sensitive reporter strain.

### tdTomato expression in the CNS of TRPA1 reporter mice

**Figure 7.**
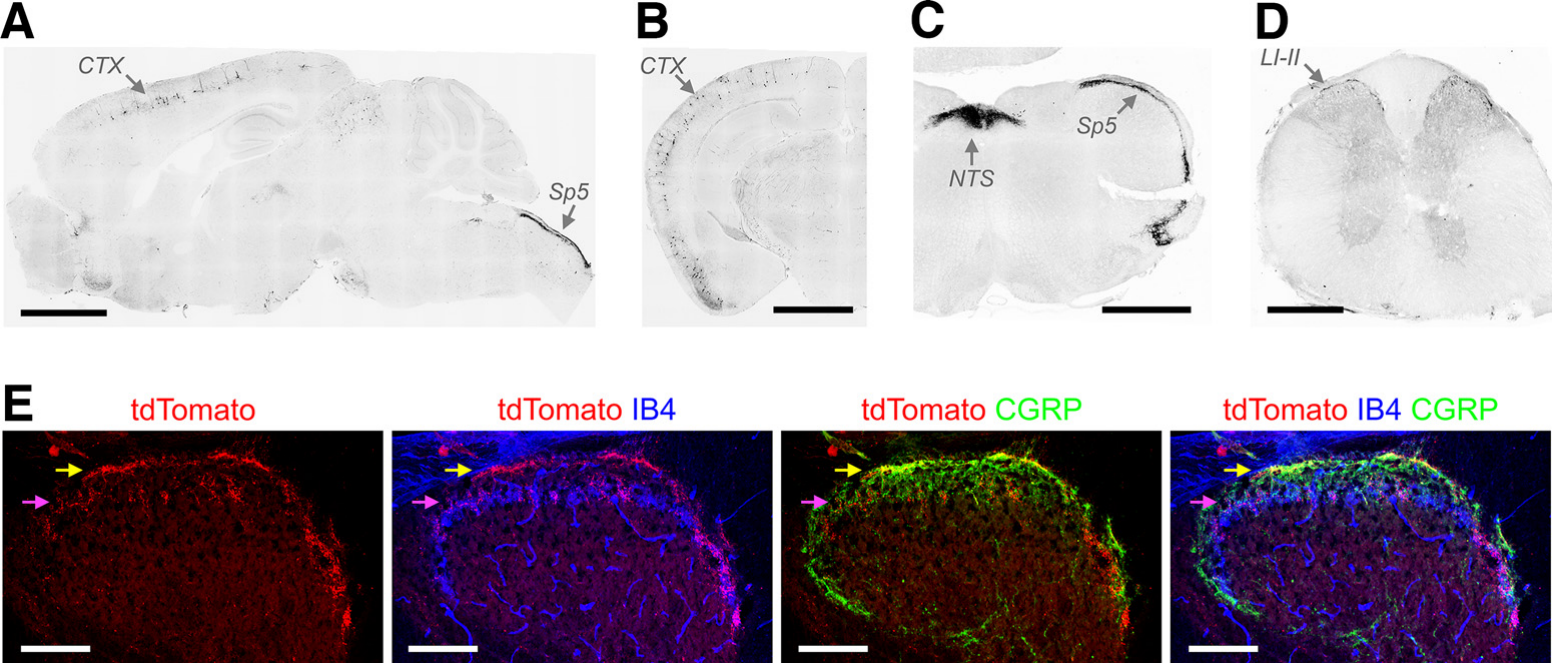
tdTomato expression within the CNS of *TRPA1^Flp^R26^ai65f^* mice. ***A***, Sagittal section of brain and brainstem showing tdTomato expression (black) in occasional neurons within the somatomotor cortex (CTX) and in fibers within the Sp5. ***B***, Coronal section of brain (2.5 mm caudal of bregma) showing tdT^+^ neurons in CTX. ***C***, Coronal section of medulla (0.4 mm caudal of obex) showing tdT^+^ fibers in the nTS and Sp5. ***D***, Coronal section of thoracic spinal cord showing tdT^+^ fibers innervating lamina I and II (LI–LII) of the dorsal horn. ***E***, High magnification of superficial lamina of the dorsal horn showing tdTomato (red), the nonpeptidergic afferent marker IB4 (blue), and the peptidergic afferent marker CGRP (green). Note that IB4 also labels some CNS blood vessels in mice (Laitinen, 1987). tdT^+^ fibers were found within the CGRP^+^ lamina (yellow arrow) and within the deeper IB4^+^ lamina (pink arrow). Scale bars: ***A***, ***B***, 2 mm; ***C***, 1 mm; ***D***, 500 μm; ***E***, 100 μm.

We investigated the expression of tdTomato in the CNS of *TRPA1^Flp/+^R26^ai65f/+^* mice (*n* = 5 mice). In the midbrain and higher brain levels, tdTomato expression was extremely limited, except for minor populations of neurons within the second/third layers of the somatomotor cortex and the piriform area (Fig. 7*A*,*B*). In the brainstem, tdTomato expression was limited to nerve fibers within the nucleus tractus solitarius (nTS) and the trigeminal complex (Sp5; Fig. 7*A*,*C*), as well as the paratrigeminal complex (Pa5; data not shown). These medullary areas receive dense innervation from vagal and trigeminal nociceptive afferents (Kim et al., 2020a). tdTomato expression in the nTS was restricted to the dorsal and medial subnuclei (e.g., SolC, SolG, SolDL). In the thoracic spinal cord, tdTomato expression was observed in nerve fibers in the dorsal horn (Fig. 7*D*). Primarily, tdTomato^+^ fibers were concentrated in the superficial laminae innervated by CGRP^+^ DRG afferents (Lamina I and, to some extent, Lamina II_o_) and by IB4^+^ DRG afferents (Lamina II_i_; Fig. 7*E*), although sporadic fibers were noted in deeper areas. We found no obvious tdTomato expression in astrocytes or glial cells in any CNS region.

### tdTomato expression in peripheral tissues of TRPA1 reporter mice

**Figure 8.**
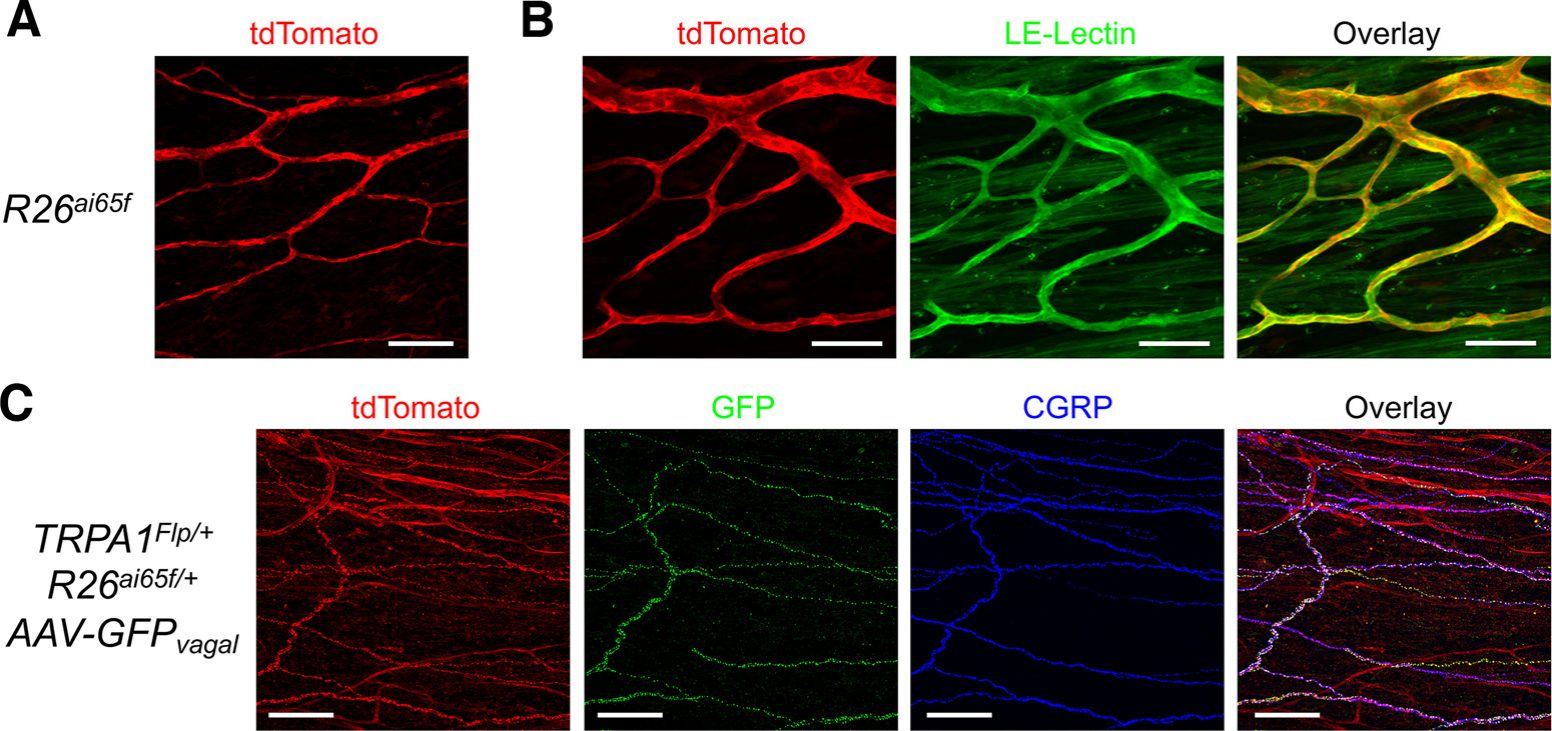
tdTomato expression in vagal afferent terminals innervating the esophageal mucosa of *TRPA1^Flp^R26^ai65f^* mice. ***A***, ***B***, tdTomato expression (red) and LE-Lectin staining (green) blood vessels within the esophagus of control *R26^ai65/ai65ff^* mice. ***C***, Esophageal mucosa of *TRPA1^Flp^R26^ai65f^* mice with vagal ganglia injection of AAV9-hSyn-GFP showing expression of tdTomato (red), GFP (green), and CGRP (blue). Scale bars: ***A***, ***C***, 100 μm; ***B***, 50 μm.

**Figure 9.**
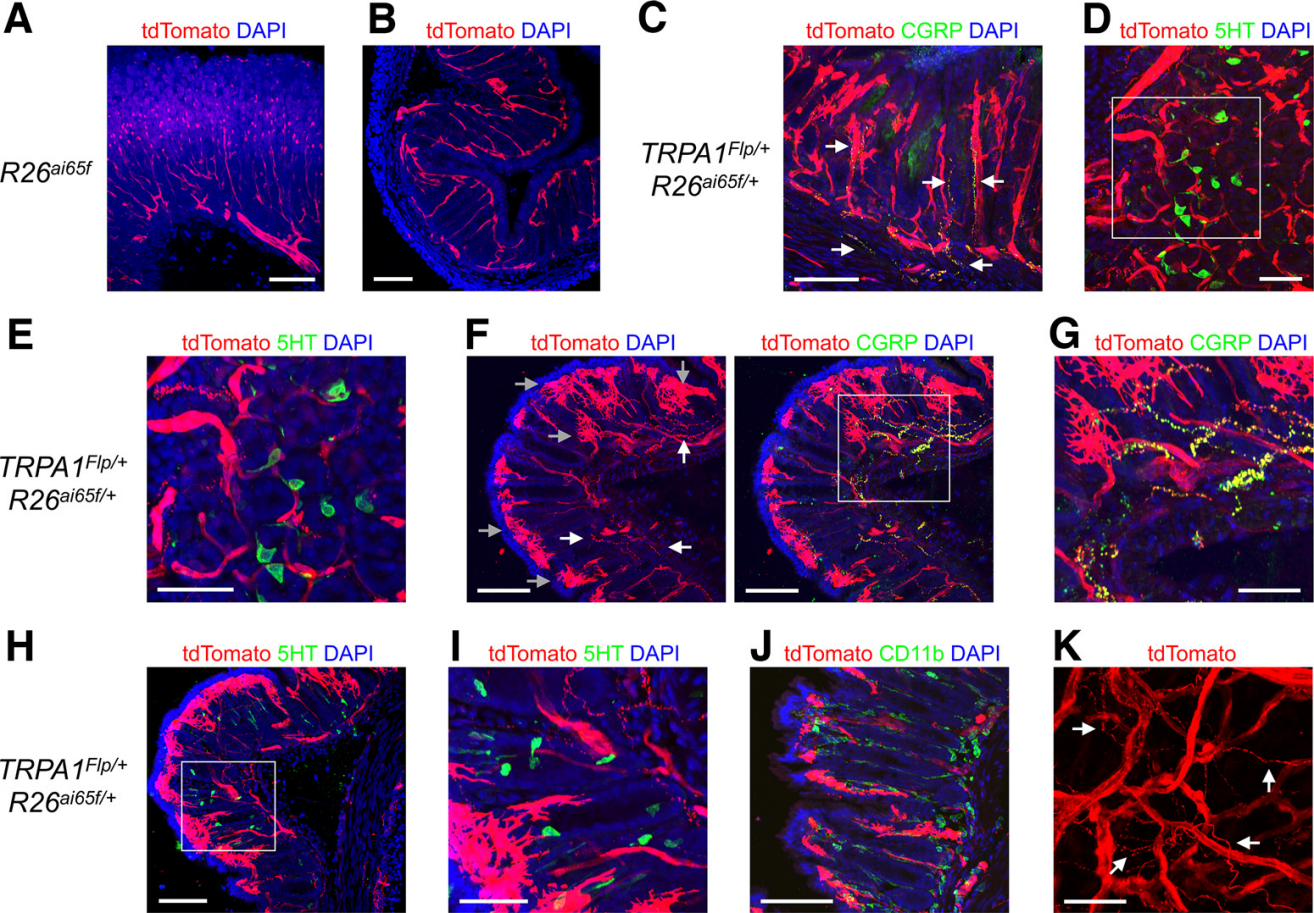
tdTomato expression in the stomach, colon, and bladder of *TRPA1^Flp^R26^ai65f^* mice. ***A***, ***B***, tdTomato expression (red) and DAPI staining (blue) in the stomach (***A***) and colon (***B***) of control *R26^ai65f/ai65f^* mice. ***C–K***, tdTomato expression (red) in tissues from *TRPA1^Flp^R26^ai65f^* mice. ***C***, Stomach with tdTomato and CGRP (green) expression and DAPI (blue). White arrows identify tdT^+^ fibers, which almost exclusively also express CGRP. ***D***, Stomach with tdTomato and 5-HT (green) expression and DAPI (blue). ***E***, Higher magnification of white box identified in ***D***. ***F***, Colon with tdTomato expression and DAPI (left), with overlay of CGRP expression (green; right). White arrows identify tdT^+^ fibers, gray arrows identify tdT^+^ stellate-shaped resident cells. ***G***, Higher magnification of white box identified in ***F***. ***H***, Colon with tdTomato and 5-HT (green) expression and DAPI (in blue). ***I***, Higher magnification of white box identified in ***H***. ***J***, Colon with tdTomato and CD11b (green) expression and DAPI (blue). ***K***, Bladder with tdTomato expression. White arrows identify tdT^+^ fibers. Scale bars: ***A***, ***B***, ***C***, ***F***, ***H***, ***J***, 100 μm; ***D***, ***E***, ***G***, ***I***, ***K***, 50 μm.

We investigated the expression of tdTomato in peripheral organs of *TRPA1^Flp/+^R26^ai65f/+^* mice and control *R26^ai65f/ai65f^* mice. We noted consistent Flp-independent tdTomato expression in many LE-lectin^+^ blood vessels within the esophagus (both muscle and mucosal layers), trachea, stomach, colon, and bladder of *R26^ai65f/ai65f^* mice (Figs. 8*A*,*B*, 9*A*,*B*; *n* = 4 mice). Immunohistochemical staining with an anti-DsRed antibody confirmed the presence of tdTomato in these control mice (data not shown). tdT^+^ blood vessels were only occasionally observed in the skin and were absent from the lungs and cochlea (data not shown). In the esophagus of *TRPA1^Flp/+^R26^ai65f/+^* mice (*n* = 6), many tdT^+^ nerve fibers were found in the mucosal and muscle layers (data not shown) along with tdT^+^ blood vessels. No tdT^+^ neuronal cell bodies were found. To determine the origin of these tdT^+^ fibers, we injected the vagal ganglia of *TRPA1^Flp/+^R26^ai65f/+^* mice with AAV9-hSyn-GFP to label the vagal afferents with GFP. There was consistent overlap of tdTomato, GFP, and CGRP staining, indicating that most tdT^+^ fibers were vagal CGRP^+^ fibers (Fig. 8*C*).

**Figure 10.**
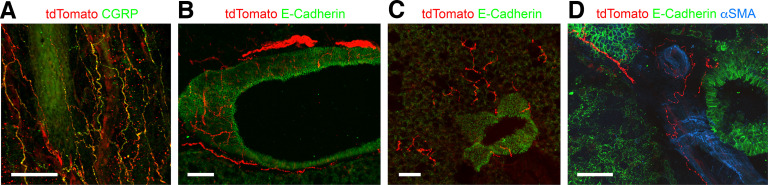
tdTomato expression in fibers innervating the trachea and lungs of *TRPA1^Flp^R26^ai65f^* mice. ***A***, tdTomato (red) and CGRP (in green) expression in the trachea (whole mount). ***B***, Lung slice showing tdT^+^ fibers (red) innervating a large conducting airway labeled by E-cadherin (green). ***C***, Lung slice showing tdT^+^ fibers (red) projecting from a small conducting airway (labeled by E-cadherin; green) into the alveolar regions. ***D***, Lung slice showing tdT^+^ fibers (red) innervating a pulmonary blood vessel that expresses α smooth muscle actin (αSMA; blue) but not E-cadherin (green). Scale bars: ***A***–***C***, 100 μm; ***D***, 50 μm.

**Figure 11.**
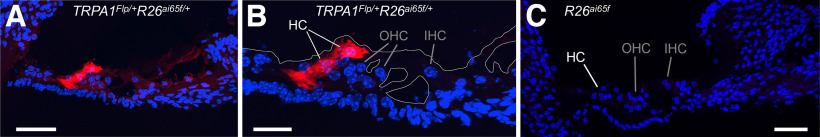
tdTomato expression in the organ of Corti in the cochlea of *TRPA1^Flp^R26^ai65f^* mice. ***A***, ***B***, tdTomato expression (red) with DAPI staining (blue) in *TRPA1^Flp^R26^ai65f^* mice. ***A***, Overexposure of tdTomato signal. ***B***, Standard exposure of tdTomato signal with cellular organization outlined with gray lines and Hensen’s cells (HCs), outer hair cells (OHCs), and inner hair cells (IHCs) identified. ***C***, Negligible tdTomato expression (red) with DAPI staining (blue) in control *R26^ai65f^* mice, with HCs, OHCs, and IHCs identified. Scale bars: ***A***, ***B***, 25 μm; ***C***, 50 μm.

In addition to the tdT^+^ blood vessels that were also observed in control *R26^ai65f/ai65f^* mice (Fig. 9*A*,*B*), tdTomato was expressed in multiple structures in the stomach and colon of *TRPA1^Flp/+^R26^ai65f/+^* mice (Fig. 9*C–J*; *n* = 4 mice). Abundant tdT^+^ fibers were found within the mucosa and the submucosal plexus. Some tdT^+^ fibers were also observed in the myenteric plexus, but these did not appear to possess intraganglionic laminar endings (IGLEs). Many tdT^+^ fibers expressed CGRP (Fig. 9*C*,*F*,*G*). No tdT^+^ neuronal cell bodies were found. Robust tdTomato expression was observed in many large stellate-shaped cells within the stomach (Fig. 9*C–E*) and colonic mucosa (Fig. 9*F*–*J*), which were absent in *R26^ai65f/ai65f^* mice (Fig. 9*A*,*B*). tdTomato expression did not overlap with either serotonin (marker for enterochromaffin cells; Bellono et al., 2017; Fig. 9*D*,*E*,*H*,*I*) or cd11b (marker for most resident macrophages and dendritic cells; Cerovic et al., 2014; Fig. 9*J*). tdTomato expression was noted in nerve fibers innervating the bladder of *TRPA1^Flp/+^R26^ai65f/+^* mice (*n* = 4 mice), as well as within some blood vessels (Fig. 9*K*). In the trachea of *TRPA1^Flp/+^R26^ai65f/+^* mice (*n* = 4), we noted dense innervation by tdT^+^ fibers, especially in the subepithelial layers of the trachealis muscle and annular ligaments (Fig. 10*A*). The tdT^+^ fibers formed a dense plexus of free nerve endings that lacked defined arbors. Almost all tdT^+^ fibers also expressed CGRP. In the lungs (*n* = 3 mice), tdT^+^ fibers densely innervated conducting airways of all diameters (Fig. 10*B–D*). Some of these tdT^+^ fibers projected out into the alveolar region (Fig. 10*C*). Some pulmonary blood vessels were also innervated by tdT^+^ fibers (Fig. 10*D*). Last, we observed tdTomato expression in the cells of Hensen in the cochlea of *TRPA1^Flp/+^R26^ai65f/+^* mice (*n* = 3; Fig. 11*A*,*B*). tdTomato expression was absent from all other cell types including inner hair cells, outer hair cells, and spiral ganglion cells. No tdTomato expression was found in the cochlea of *R26^ai65f/ai65f^* mice (*n* = 2; Fig. 11*C*).

### Chemogenetic activation of TRPA1-expressing nerves in the paw evokes nocifensive behaviors

**Figure 12.**
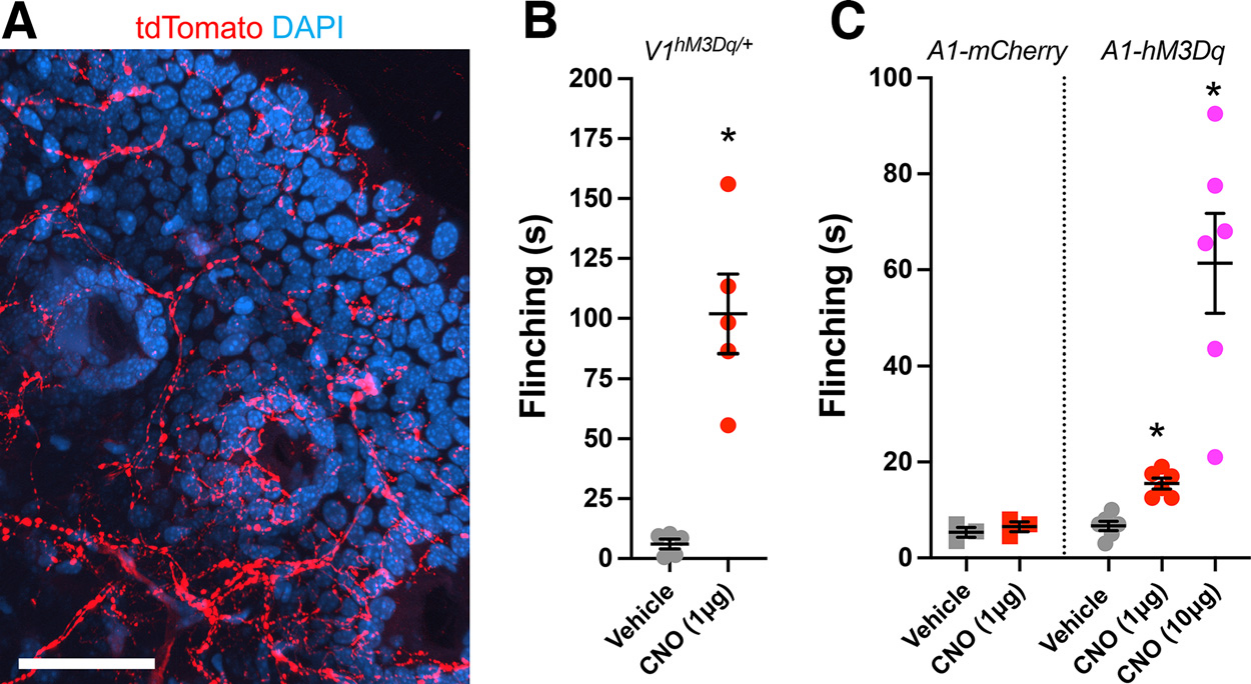
Chemogenetic activation of TRPA1-expressing nerves in the hindpaw evokes nocifensive behavior. ***A***, tdTomato expression (red) in fibers innervating the hairy skin of the hindpaw of *TRPA1^Flp^R26^ai65f^* mice. Scale bar, 50 μm. ***B***, Mean ± SEM flinching time of TRPV1-hM3Dq mice following intraplantar injection of vehicle or CNO (1 μg). ***C***, Mean ± SEM flinching time following intraplantar injection of vehicle or CNO (1 or 10 μg) in *TRPA1^Flp^* mice previously injected with rAAV2-fDIO-hM3Dq (A1-hM3Dq) or with control rAAV2-fDIO-mCherry (A1-mCherry). *Significant difference in flinching time compared with vehicle (*p* < 0.05).

We observed dense tdT^+^ innervation of hairy skin and glabrous skin on the hindpaw from *TRPA1^Flp/+^R26^ai65f/+^* mice (Fig. 12*A*). tdTomato was expressed by free nerve endings (that lacked defined arbors) within the dermis and epidermis. We did not observe tdTomato expression in other cell types, including keratinocytes. To determine the effect of the activation of specific afferent populations within the skin on nocifensive/pain behaviors including flinching, we expressed the CNO-sensitive stimulatory DREADD receptor hM3Dq in TRPV1-expressing and TRPA1-expressing nerves. First, we found that intraplantar injection of CNO (1 μg) into the hindpaw of TRPV1-hM3Dq mice evoked a significant increase in flinching time compared with vehicle (*p* < 0.05, *n* = 5 mice; Fig. 12*B*). To drive hM3Dq expression in TRPA1-expressing nerves, we injected the hindpaw of *TRPA1^Flp/Flp^* mice with rAAV2-fDIO-hM3Dq (or rAAV2-fDIO-mCherry as a negative control). Four weeks later, we performed intraplantar injection of the hindpaw with vehicle, 1 μg of CNO, and 10 μg of CNO. In the TRPA1-hM3Dq mice (*n* = 6), CNO at both concentrations evoked a significant increase in flinching time compared with vehicle (*p* < 0.05; Fig. 12*C*). Importantly, CNO had no effect on flinching time compared with vehicle in the control TRPA1-mCherry mice (*p* > 0.05, *n* = 3; Fig. 12*C*).

## Discussion

We developed the *TRPA1^Flp^* mouse as a tool to detect and selectively manipulate TRPA1-expressing cells. Although selective TRPA1 agonists and antagonists have been extensively reported, there is disagreement in the literature regarding the cellular expression patterns of TRPA1 in mammals—especially in nonafferent cell types—in part because of the inconsistent/poor antibodies available. Here we used a genetic approach, replacing the endogenous TRPA1 stop codon with a 2A-Flp_O_ cassette. As 2A is a self-cleaving peptide, we expect that the expression of Flp recombinase would match TRPA1 peptide expression on a one-to-one basis. Crossing the *TRPA1^Flp^* mouse with Flp-sensitive reporter mice (e.g., *R26^ai65f^*) is then a powerful tool to visualize TRPA1 expression.

TRPA1 is a nonselective cation channel that is activated by reactive oxygen species (e.g., H_2_O_2_), electrophilic products of lipid peroxidation (e.g., 4-hydroyxynonenal), and electrophilic irritants (e.g., AITC, cinnamaldehyde, and acrolein; Bandell et al., 2004; Jordt et al., 2004; Bautista et al., 2006; Hinman et al., 2006; Andersson et al., 2008; Taylor-Clark et al., 2008a). As such, TRPA1 signaling has been implicated in chemosensitivity, oxidative stress, and inflammation (Wu et al., 2010). TRPA1 expression, as assessed functionally and by transcript analysis, is well established in a subset of sensory afferent neurons within the dorsal root, and vagal and trigeminal ganglia (Story et al., 2003; Jordt et al., 2004; Nassenstein et al., 2008), and the administration of TRPA1 agonists evokes pain and other nocifensive reflexes in a TRPA1-dependent manner (Bautista et al., 2006; Kwan et al., 2006; McNamara et al., 2007; Bessac et al., 2008; Taylor-Clark et al., 2009; Liu et al., 2013; Patil et al., 2020). Nevertheless, most studies characterizing TRPA1 expression have focused on individual neurons in acute cultures following enzymatic dissociation, which can have profound effects on transcript expression and electrophysiological properties (Ono et al., 2012; Song et al., 2018). The precise identity of afferents that express TRPA1 *in vivo*, particularly in relation to their coexpression of other receptors (e.g., TRPV1, MrgprA3, MrgprD) and neurotransmitters (e.g., substance P, CGRP), is debated (see below).

In the vagal ganglia of *TRPA1^Flp/+^R26^ai65f/+^* mice, we observed tdTomato expression in a major subset (∼40%) of both nodose and jugular ganglionic neurons, consistent with previous reports of their sensitivity to TRPA1 agonists (Andrè et al., 2008; Nassenstein et al., 2008; Taylor-Clark et al., 2008b; Lin et al., 2015; Stanford et al., 2019). Most of these neurons also expressed the canonical nociceptive receptor TRPV1 and the neuropeptide CGRP, indicating that they were likely nociceptive in nature. Approximately 90% of dissociated tdT^+^ vagal neurons expressed TRPA1 mRNA and were activated by the selective agonist AITC, confirming the effectiveness of the reporter. Interestingly, ∼25% of dissociated tdT^–^ vagal neurons also had TRPA1 expression/functionality, but this was much lower for tdT^–^ neurons within the intact vagal ganglion (∼7%). It is possible that dissociation selectively depletes tdTomato protein within the soma or otherwise renders tdTomato less detectable, although no evidence of this was found in previous studies of *TRPV1^Cre^R26^ai9^* mice or *P2X_2_^Cre^R26^ai9^* neurons (Stanford et al., 2019; Kim et al., 2020b). Alternatively, acute dissociation and culture may have caused *de novo* TRPA1 expression in a subset of TRPV1^+^ vagal neurons, possibly because of neurotrophic factors present in the FBS (Diogenes et al., 2007; Ciobanu et al., 2009). Importantly, AITC activated ∼40% of dissociated vagal neurons—similar to the percentage of neurons expressing tdTomato observed in frozen sections of the vagal ganglia—arguing against dissociation-evoked *de novo* TRPA1 expression. Indeed, native tdTomato expression in vagal ganglia sections was only detectable in some neurons after signal augmentation using antibodies suggesting some native fluorescence was near the limits of detection.

Given that tdTomato expression denotes Flp-mediated recombination of the *R26^ai65f^* locus at any time, the strong correlation of tdTomato with TRPA1 expression suggests that very few vagal neurons transiently express TRPA1 during development.

Some studies have identified a small subset of TRPA1^+^TRPV1^–^ vagal neurons in functional (Nassenstein et al., 2008; Taylor-Clark et al., 2008b; Stanford et al., 2019) and transcript studies (Mazzone et al., 2020) of dissociated neurons. Other studies of transcripts in dissociated neurons (Nassenstein et al., 2008; Brozmanova et al., 2011; Kupari et al., 2019; Meerschaert et al., 2020; Zhao et al., 2022), and single fiber recordings of vagal afferents innervating the airways or esophagus (Nassenstein et al., 2008; Brozmanova et al., 2011; Nesuashvili et al., 2013) argue that TRPA1 expression is confined to a subset of TRPV1^+^ vagal neurons. Here, we found ∼20% of tdT^+^ neurons in vagal sections lacked immunoreactivity to TRPV1. However, we detected TRPV1 transcript in all tdT^+^ vagal neurons and found that >95% of these neurons were capsaicin-sensitive. While there are caveats to interpreting TRP expression in dissociated cultures, it is probable that the high count of tdT^+^TRPV1^–^ neurons in vagal sections is partly because of inefficiencies of the TRPV1 antibody used in the present study.

tdTomato was expressed in only 3–12% of neurons within the DRGs of *TRPA1^Flp/+^R26^ai65f/+^* mice. Most of these neurons also expressed TRPV1 and CGRP. Following dissociation, ∼85% of tdT^+^ DRG neurons were activated by AITC compared with ∼25% of tdT^–^ vagal neurons. Given that few DRG neurons expressed tdTomato, this meant that most AITC-sensitive neurons were tdT^–^. This did not appear to be because of culture-induced *de novo* TRPA1 expression because similar percentages (35–45%) of DRG neurons in intact ganglia and dissociated cultures were activated by AITC. Thus, our TRPA1 reporter preferentially labels TRPA1-expressing DRG neurons but with low efficiency. Functional and transcript studies have shown that TRPA1 expression in the DRG correlates with TRPV1 expression. In addition to the expected tdT^+^TRPV1^+^ population, our data also suggest evidence of a TRPA1^+^TRPV1^–^ subset in DRGs, and this is consistent with some reports (Malin et al., 2011; Usoskin et al., 2015; Patil et al., 2018) whereas others argue that TRPA1 is expressed exclusively in TRPV1^+^ neurons (Story et al., 2003; Mishra et al., 2011; Pogorzala et al., 2013; Wilson et al., 2013; Weng et al., 2015). The tdT^+^TRPV1^–^ DRG neurons observed here were larger than tdT^+^TRPV1^+^ DRG neurons but were much smaller than tdT^–^TRPV1^–^ neurons, which are likely to be non-nociceptive mechanoreceptors.

The limited reporter efficiency in the DRGs of *TRPA1^Flp/+^R26^ai65f/+^* mice was recapitulated in *TRPA1^Flp/+^R26^FLTG/+^* mice, implicating the Flp itself. Here we used Flp_O_, which is the thermostable Flp_E_ recombinase enzyme whose nucleotide sequence has been optimized for mammalian codons (Raymond and Soriano, 2007). When expressed in HEK293 cells, Flp_O_ recombines FRT sites as efficiently as Cre recombines LoxP sites (Fenno et al., 2014). Although only four Flp molecules are technically necessary for recombination of paired FRT sites, the reduced efficiency of Flp_E_ compared with Flp_O_ (which are identical proteins) indicates that physiologically relevant expression levels are rate limiting (Raymond and Soriano, 2007; Kranz et al., 2010). Thus, it is possible that the low but functionally relevant expression of TRPA1 in some TRPA1^+^ DRG neurons fails to drive sufficient Flp expression to recombine the ROSA26 FRT sites for tdTomato expression. Indeed, the AITC-evoked responses of AITC-sensitive neurons was smaller in tdT^–^ than in tdT^+^ DRG neurons, suggesting lower TRPA1 expression. Nevertheless, we noted that even when *R26^ai65f^* tdTomato expression was evoked in sensory afferents (as observed in frozen sections), its detection more often required antibody-mediated signal augmentation in DRG tdT^+^ neurons (28.9%) compared with vagal tdT^+^ neurons (8.1%), suggesting lower tdTomato expression per neuron in DRGs independent of the efficiency of Flp recombination.

Comprehensive direct comparisons between Cre- and Flp-expressing mouse strains are limited (Daigle et al., 2018), but one study of corticotropin-releasing hormone (CRH) reporter strains showed reduced efficiency of Flp_O_ recombination of *R26^ai65f/+^* compared with Cre recombination of *R26^ai9/+^* (Zhao et al., 2023): while the two strategies labeled identical CNS regions, more tdT^+^ neurons were noted for the Cre reporter because it was more efficient evoking tdTomato expression in neurons expressing lower levels of CRH. Interestingly, the efficiency of reporting was increased using homozygous *CRH^Flp^* mice. It is possible that more TRPA1-expressing DRG neurons would express tdTomato using homozygous *TRPA1^Flp^* mice.

We used the *TRPA1^Flp/+^R26^ai65f/+^* mouse to map TRPA1-expressing afferents. We observed dense innervation of the gut, bladder, skin, and airways by tdT^+^ fibers, but no intrinsic tdT^+^ neurons were found in these organs. Most of the tdT^+^ fibers innervating the esophagus mucosa were derived from the vagal ganglia. Abundant tdT^+^ fibers were also found within the mucosa and the submucosal plexus of the stomach and colon. Many tdT^+^ fibers in the gut expressed CGRP, but no evidence was found of tdT^+^ fibers with IGLEs. In the lungs, we observed tdT^+^ fibers innervating conducting airways and blood vessels, similar to vagal TRPV1^+^ fibers previously described (Su et al., 2021; Kim et al., 2022). The observation that some of the tdT^+^ fibers here also penetrated the alveolar space argues that these were projected from nodose neurons (Kim et al., 2022). In the skin, we found abundant tdT^+^-free nerve endings within the dermis and epidermis that resembled TRPV1^+^ and CGRP^+^ fibers (Cavanaugh et al., 2011; McCoy et al., 2012; Le Pichon and Chesler, 2014). In the brainstem medulla, abundant tdT^+^ fibers were found terminating in the nTS, Pa5, and Sp5, consistent with the innervation patterns of vagal and trigeminal TRPV1^+^ fibers (Kim et al., 2020a; Su et al., 2021). In the spinal cord, tdT^+^ fibers mostly innervated dorsal horn lamina I and II, mimicking previous studies of nociceptive peptidergic and nonpeptidergic Aδ- and C-fibers projected from DRG neurons (Cavanaugh et al., 2011; McCoy et al., 2012; Le Pichon and Chesler, 2014) that synapse with spinal neurons involved in pain processing (Todd, 2010). AITC has previously been shown to evoke glutamate-mediated excitatory postsynaptic potentials in nTS and dorsal horn neurons, implicating the presence of TRPA1-expressing presynaptic terminals (Kosugi et al., 2007; Sun et al., 2009).

Despite the relatively low efficiency of the Flp reporter in DRGs, chemogenetic activation of TRPA1-expressing afferents innervating the hindpaw evoked flinching, similar in quality to chemogenetic activation of TRPV1-expressing afferents (albeit at lower intensity). Such responses are consistent with the expression of TRPA1 in nociceptive DRG afferents, and multiple studies showing TRPA1 agonists evoke pain (Bautista et al., 2006; Kwan et al., 2006; McNamara et al., 2007; Patil et al., 2020).

TRPA1 was originally cloned from cultured human fibroblasts (Jaquemar et al., 1999), and TRPA1 expression has subsequently been reported in multiple nonafferent cell types, including hair cells in the cochlea (Corey et al., 2004), keratinocytes (Atoyan et al., 2009; Kwan et al., 2009), airway epithelium and smooth muscle cells (Nassini et al., 2012; Kannler et al., 2018), enterochromaffin cells (Cho et al., 2014; Bellono et al., 2017), cerebral artery endothelium (Earley et al., 2009), astrocytes (Shigetomi et al., 2012; Takizawa et al., 2018), Schwann cells (De Logu et al., 2017; 2019), and cortical and hippocampal neurons (Koch et al., 2011; Kheradpezhouh et al., 2017). The determination of TRPA1 expression in these cell types was often controversial when based on low-specificity techniques (e.g., antibodies) lacking supporting evidence, especially given other reports that TRPA1 transcript expression was very limited in rodents (Story et al., 2003; Nagata et al., 2005; Jang et al., 2012; Reese et al., 2020).

The use of our *TRPA1^Flp^* reporter to map TRPA1 expression was somewhat hampered by the Flp-independent expression of tdTomato in *R26^ai65f^* mice blood vessels, particularly in the gut and bladder, partially in the skin and trachea and virtually absent in the lung and CNS. FRT-stop-FRT elements (which precedes the tdTomato sequence in the ROSA26 knockin) are not completely “leak proof” (Dabrowski et al., 2015), and thus may account for the tdTomato expression in these blood vessels. We are not aware of any other previous reports that investigate peripheral tissues in *R26^ai65f^* mice, but Flp-independent tdTomato expression does not appear to occur in the CNS (Yao et al., 2020).

We found no expression of tdTomato, other than in afferent-like fibers and some blood vessels, in the skin, trachea, or lungs of *TRPA1^Flp/+^R26^ai65f/+^* mice, and thus we can provide no evidence for TRPA1 expression in keratinocytes, airway epithelium, or airway smooth muscle cells. We found many large tdT^+^ stellate-shaped cells within the stomach and colonic mucosa; however, these expressed neither serotonin nor cd11b, indicating that they were neither enterochromaffin cells nor resident macrophages/dendritic cells, respectively. The role of TRPA1 in the function of these unidentified cells is presently not known. We found tdTomato expression in a small subset of cortical neurons within the second/third layers of the somatomotor cortex and the piriform area, but no tdTomato expression was found in astrocytes, Schwann cells, or vascular endothelium. Last, in the cochlea we observed robust tdTomato expression in Hensen’s cells, which are members of a group of supporting cells for the inner and outer hair cells in the organ of Corti, but not in the hair cells themselves. Initial reports using antibodies reported TRPA1 expression in inner hair cells (Corey et al., 2004; Nagata et al., 2005), but the *in situ* hybridization signal (Corey et al., 2004) appeared adjacent to the outer hair cells—consistent with the location of Hensen’s cells. Recently, AITC-evoked Ca^2+^ transients have been shown in Hensen’s cells (Vélez-Ortega et al., 2023).

Overall, we find limited expression of tdTomato in nonafferent cell types in *TRPA1^Flp/+^R26^ai65f/+^* mice. While the inhibition or knockout of TRPA1 reduces nociceptive responses to electrophilic irritants, oxidative stress, and inflammatory pain models (Bautista et al., 2006; Kwan et al., 2006; Dai et al., 2007; McNamara et al., 2007; Eid et al., 2008), a role for TRPA1 has also been reported for other physiological and pathophysiological processes such as endothelium-dependent cerebral artery dilation (Earley et al., 2009), anxiety (de Moura et al., 2014), cognition and memory (Lee et al., 2016a,b), which are unlikely to be mediated by sensory nerves. It is possible that these are because of off-target effects of TRPA1 knockout/inhibitors. Alternatively, it is possible that some tdT^–^ cells in our reporter express functionally relevant levels of TRPA1 without sufficient Flp expression to drive tdTomato expression. Finally, *de novo* expression of TRPA1 in nonafferent cell types under pathologic conditions (not tested here) could explain the role of TRPA1 in some processes.

In summary, we describe a *TRPA1^flp^* mouse that provides a new tool for the selective identification and manipulation of TRPA1-expressing cells. Our data confirm that TRPA1 is expressed by a subset of sensory neurons, which largely but not exclusively overlap with the canonical nociceptive TRPV1 channel. We also find limited TRPA1 expression in other cell types, but most notably in subepithelial cells in the mucosa of the stomach and colon. Recently, a TRPA1^cre^ mouse line has been reported (Matsuo et al., 2021). When crossed with a Cre-sensitive enhanced yellow fluorescent protein (YFP) expressing mouse, YFP^+^ neurons were identified in vagal and trigeminal ganglia, with some YFP^+^ fibers innervating the nTS and Sp5 regions in the medulla, consistent with our findings here.

## Synthesis

Reviewing Editor: Christopher Colwell, UCLA School of Medicine Decisions are customarily a result of the Reviewing Editor and the peer reviewers coming together and discussing their recommendations until a consensus is reached. When revisions are invited, a fact-based synthesis statement explaining their decision and outlining what is needed to prepare a revision will be listed below. The following reviewer(s) agreed to reveal their identity: Sven-Eric Jordt, Stuart Bevan.

This manuscript helps resolving a long-standing controversy about the tissue distribution of TRPA1, a pain-transducing ion channel primarily expressed in peripheral somatosensory neurons, but also claimed to be expressed in other tissues such as brain, lung epithelial cells, skin and intestine. The authors clearly show that TRPA1 expression is restricted to sensory neurons, with the intestinal epithelium the only other site of local expression. It also demonstrates that TRPA1 expressing neurons are nociceptors, by chemogenetic targeting. These data are highly important, especially in the background of ongoing clinical trials using TRPA1 inhibitors.

In this manuscript, the authors report the creation of a new TRPA1Flp/+R26ai65f/+ mouse line as a powerful new tool for the selective identification and manipulation of TRPA1-expressing cells in vivo. The data in this manuscript confirm that TRPA1 is expressed by a subset of sensory neurons, largely overlapping with neuron expressing the nociceptive TRPV1 channel, and there is minimal or absent TRPA1 expression in other cell types. These findings help to clarify the current debate regarding the cellular expression of TRPA1 in mammals. The authors also demonstrate that chemogenetic activation of TRPA1-expressing nerves in the paw evoked flinching behavior.

This well-presented study also clearly describes the development of a TRPA1Flp reporter mouse and its crossing with a reporter strain (R26ai65f) in order to identify TRPA1 expression and tissue distribution. The experimental approach is well designed and the paper is well organized and written clearly. The positive and negative controls for each experiment are well conceived and the data convincingly support the authors’ conclusions.

Still the reviewers found some issues that need to be addressed. In particular, the data presented raise concerns about the limitations of tdTomato fluorescence to monitor TRPA1 expression accurately in all tissues, which could be reflected better in a narrative focused on the strengths and weaknesses of this reporter mouse line.

Main points to be addressed:

1. The authors cite an appropriate balance of recent and older references, however, there are several key references missing that report TRPA1 expression profiling experiments of relevance for this manuscript, some comparing expression between sensory neurons and the tissues investigated in this study :

- Reese, R.M., Dourado, M., Anderson, K. et al. “Behavioral characterization of a CRISPR-generated TRPA1 knockout rat in models of pain, itch, and asthma”. Sci Rep 10, 979 (2020). https://doi.org/10.1038/s41598-020-57936-5

- Cho, HJ., Callaghan, B., Bron, R. et al. “Identification of enteroendocrine cells that express TRPA1 channels in the mouse intestine”. Cell Tissue Res 356, 77-82 (2014). https://doi.org/10.1007/s00441-013-1780-x

- Jang, Y., Lee, Y., Kim, S.M. et al. “Quantitative analysis of TRP channel genes in mouse organs”. Arch. Pharm. Res. 35, 1823-1830 (2012). https://doi.org/10.1007/s12272-012-1016-8

- Liu B., Escalera J., Balakrishna S. et al. “ TRPA1 controls inflammation and pruritogen responses in allergic contact dermatitis”. FASEB J. 2013 Sep;27(9):3549-63. https://doi.org/10.1096/fj.13-229948

2. Different from guinea pig and larger mammals, nodose and jugular ganglia in mice are hard to distinguish, merged into one jugular/nodose complex (vagal ganglion). However, the authors state, “The vagal ganglion is comprised of the anatomically distinct nodose ganglion and the jugular ganglion”, and perform a detailed analysis of neurons from both ganglia separately in Fig. 3. Please, explain how both ganglia are anatomically distinct in

the mouse and how the discrimination between jugular and nodose ganglion was achieved.

3. According to the authors as well, and as shown in Fig. 4, “based upon widespread expression of P2X2 and the limited expression of Tac1, it is likely that most of these vagal neurons were derived from the nodose ganglia”. However, Fig.3 compares TRPA1 and TRPV1 expression in nodose neurons and in jugular neurons. Please, explain how the differentiation between both types of ganglionic neurons was performed for Fig.3.

4. Is there any indication of reporter expression in Schwann cell ? Recently, several studies reported expression of TRPA1 in Schwann cells, with Schwann cell TRPA1 essential for the development of certain forms of pain (PMID: 31487269, 29192190)

5. A comparison of the native tdTomato fluorescence with immunostaining for tdTomato shows that the native tdTomato fluorescence reports expression in ∼90% of vagal neurons but in only ∼71% of DRG neurons, suggesting weaker tdTomato expression in DRG neurons. This would fit with other data on tdTomato expression and indicates that in some cells the lack of a native tdTomato signal can be ascribed to insufficient sensitivity to detect low levels of tdTomato expression/fluorescence. No details of the microscope system used for the functional studies are given.

6. A notable feature is the mismatch in the expression of tdTomato and AITC sensitivity which the authors suggest is due to a) de novo gain of AITC sensitivity for vagal neurons but b) weak tdTomato expression for DRG neurons. The evidence for de novo synthesis in tdTomato-negative vagal neurons is not compelling. Experiments with intact vagal ganglia showed that ∼96% of neurons responding to the TRPA1 agonist AITC were tdTomato-positive and only 7.6% of tdTomato-negative neurons responded to AITC. However, while most (∼91%) dissociated tdTomato-positive neurons responded to AITC, 23% of tdTomato-negative vagal neurons responded to AITC. Although this would fit with a ‘gain-of-function’ in some neurons it could be explained by weaker tdTomato expression after dissociation as the percentage of tdTomato-positive neurons was ∼40% in intact vagal ganglia but only 24% in dissociated cell culture. This could match with the finding of smaller amplitude AITC-evoked responses in tdTomato-negative than tdTomato-positive neurons. In addition, the percentage of AITC -responsive neurons in intact ganglia (∼60%, 128/210) is higher than for dissociated cells (∼50%, 92/189), which is not consistent with de novo expression of TRPA1 after dissociation.

7. The mismatch between tdTomato staining and AITC sensitivity in thoracic DRG neurons is marked. Only 12.5% of DRG neurons in intact ganglia expressed detectable tdTomato but this was reduced to 5.5% in dissociated cell culture, again suggesting reduced tdTomato expression after dissociation. In contrast, ∼32% of the neurons in culture responded to AITC, which is similar to the percentage of responsive neurons (37%) recorded in intact GCamp6 ganglia. As stated by the authors the low percentage of tdTomato-positive neurons may be due to inefficient tdTomato expression in DRG neurons but the sensitivity required to detect tdTomato fluorescence may also be a significant factor.

8. The study also examines tdTomato expression in the CNS and several peripheral tissues where TRPA1 expression has previously been reported. This section of the manuscript raises some other concerns. Studies with control R26ai65f/ai65f mice show that Flp-independent expression of tdTomato can occur in some but not all blood vessels. It is not clear that control R26ai65f/ai65f mice were studied to check whether the tdTomato staining seen in other non-sensory nerve structures in the stomach, colon and cochlea were Flp-dependent or - independent and therefore related to TRPA1 expression. These data should be included. Furthermore, the lack of tdTomato fluorescence in other cells particularly those previously reported to `express TRPA1 could simply reflect undetectable, low levels of tdTomato expression in these cells as found in many DRG neurons.

9. In the discussion, the authors suggest some reasons for the failure to detect tdTomato fluorescence in every cell. Studies with the second reporter line generated (TRPA1Flp/+R26FLTG/+) demonstrate that this is not dependent on the Flp-sensitive reporter. The authors speculate that higher expression levels may be attained by using homozygous TRPA1FLP mice or by using Cre rather than Flp reporter mice. The sensitivity required to detect the fluorescence should also be discussed. The antibody staining study with DRG neurons showed that tdTomato expression is not always evident from the native dtTomato fluorescence.

Minor Points:

1. Animal Use: The authors mention that both male and female mice were used for the experiments. Please clarify if they were homogenously distributed and used in all the experiments. Were the slices / cells a pool or combination from male and female?

2. The in vivo experiment analyzing the nocifencive response elicited by chemogenetic activation of TRPA1-expressing nerves the number of mice per group vary between 3 and 6. Please, indicate if they were male and/or female and how many times the experiment was performed (Fig. 12).

3. Review subject-verb agreement: Line 205 (trachea were instead of was); Line 276 (Changes... was instead of were); Line 352 (time... were instead of was)...

4. Line 149: Substitute Glaborus by glabrous.

5. Line 154: The trachea, esophagus and... (add “lungs”)

## Author Response

Authors’ statement: We thank the reviewers and editor for their helpful comments and have addressed each point in detail below.

Main points to be addressed:

1. The authors cite an appropriate balance of recent and older references, however, there are several key references missing that report TRPA1 expression profiling experiments of relevance for this manuscript, some comparing expression between sensory neurons and the tissues investigated in this study :

- Reese, R.M., Dourado, M., Anderson, K. et al. “Behavioral characterization of a CRISPR-generated TRPA1 knockout rat in models of pain, itch, and asthma”. Sci Rep 10, 979 (2020). https://doi.org/10.1038/s41598-020-57936-5

- Cho, HJ., Callaghan, B., Bron, R. et al. “Identification of enteroendocrine cells that express TRPA1 channels in the mouse intestine”. Cell Tissue Res 356, 77-82 (2014). https://doi.org/10.1007/s00441-013-1780-x

- Jang, Y., Lee, Y., Kim, S.M. et al. “Quantitative analysis of TRP channel genes in mouse organs”. Arch. Pharm. Res. 35, 1823-1830 (2012). https://doi.org/10.1007/s12272-012-1016-8

- Liu B., Escalera J., Balakrishna S. et al. “ TRPA1 controls inflammation and pruritogen responses in allergic contact dermatitis”. FASEB J. 2013 Sep;27(9):3549-63. https://doi.org/10.1096/fj.13-229948

Authors’ response: We appreciate the suggestion and have added these references to the text.

2. Different from guinea pig and larger mammals, nodose and jugular ganglia in mice are hard to distinguish, merged into one jugular/nodose complex (vagal ganglion). However, the authors state, “The vagal ganglion is comprised of the anatomically distinct nodose ganglion and the jugular ganglion”, and perform a detailed analysis of neurons from both ganglia separately in Fig. 3. Please, explain how both ganglia are anatomically distinct in the mouse and how the discrimination between jugular and nodose ganglion was achieved.

Authors’ response: We agree that the mouse nodose and jugular ganglia are partially fused. Nevertheless, our experience with reporter mice that selectively label nodose vs jugular neurons (e.g. P2X2-cre, 5HT3-cre, Wnt1-cre, PMID: 32669344, 32060036, 20937710) shows that in almost all cases the anatomical shape of the ganglia can be divided by eye into nodose and jugular portions:

The figure shows slices from 5 different vagal ganglia from P2X2Cre/R26ai9 mice, which expresses tdTomato (in red) in placode-derived neurons - i.e. nodose but not jugular neurons. In each case the bulbous nodose and the less structured jugular can be anatomically separated by eye. This data is from Kim et al 2020 (PMID: 32669344).

We acknowledge that this is not 100% effective, but we believe this approach is sufficient to quantify expression of markers in nodose vs jugular populations. We have modified the text to better clarify the ‘approximate’ nature of this approach.

3. According to the authors as well, and as shown in Fig. 4, “based upon widespread expression of P2X2 and the limited expression of Tac1, it is likely that most of these vagal neurons were derived from the nodose ganglia”. However, Fig.3 compares TRPA1 and TRPV1 expression in nodose neurons and in jugular neurons. Please, explain how the differentiation between both types of ganglionic neurons was performed for Fig.3.

Authors’ response: Fig 3 refers to frozen slices of the vagal ganglia, with the nodose and jugular portions of the ganglia visually discriminated by the anatomic shape of the vagal ganglia (see discussion above). To increase transparency, we have modified Figure 3 to show the entire vagal ganglia in 3A - demonstrating this approach. Fig 4 refers to a different data set: we dissociated the entire vagal ganglion and picked individual neurons for single cell RT-PCR. We used the detection of transcripts for P2X2 as a positive marker for nodose neurons.

4. Is there any indication of reporter expression in Schwann cell ? Recently, several studies reported expression of TRPA1 in Schwann cells, with Schwann cell TRPA1 essential for the development of certain forms of pain (PMID: 31487269, 29192190)

Authors’ response: We found no evidence of tdTomato expression in Schwann cells. This has been added to the text.

5. A comparison of the native tdTomato fluorescence with immunostaining for tdTomato shows that the native tdTomato fluorescence reports expression in ∼90% of vagal neurons but in only ∼71% of DRG neurons, suggesting weaker tdTomato expression in DRG neurons. This would fit with other data on tdTomato expression and indicates that in some cells the lack of a native tdTomato signal can be ascribed to insufficient sensitivity to detect low levels of tdTomato expression/fluorescence. No details of the microscope system used for the functional studies are given.

Authors’ response: We have clarified in the methods that the Fura2AM studies were performed on a Nikon Eclipse Ti inverted microscope with a CFI Super Fluor 10x objective using a CoolSnap HQ2 camera and Nikon Elements software. We agree that native tdTomato fluorescence may undetectable in some cells - this is discussed further below.

6. A notable feature is the mismatch in the expression of tdTomato and AITC sensitivity which the authors suggest is due to a) de novo gain of AITC sensitivity for vagal neurons but b) weak tdTomato expression for DRG neurons. The evidence for de novo synthesis in tdTomato-negative vagal neurons is not compelling. Experiments with intact vagal ganglia showed that ∼96% of neurons responding to the TRPA1 agonist AITC were tdTomato-positive and only 7.6% of tdTomato-negative neurons responded to AITC. However, while most (∼91%) dissociated tdTomato-positive neurons responded to AITC, 23% of tdTomato-negative vagal neurons responded to AITC. Although this would fit with a ‘gain-of-function’ in some neurons it could be explained by weaker tdTomato expression after dissociation as the percentage of tdTomato-positive neurons was ∼40% in intact vagal ganglia but only 24% in dissociated cell culture. This could match with the finding of smaller amplitude AITC-evoked responses in tdTomato-negative than tdTomato-positive neurons. In addition, the percentage of AITC -responsive neurons in intact ganglia (∼60%, 128/210) is higher than for dissociated cells (∼50%, 92/189), which is not consistent with de novo expression of TRPA1 after dissociation.

Authors’ response: We had only compared the amplitude of AITC-evoked responses in AITC-sensitive neurons in the tdT+ and tdT- dissociated DRG populations. Identical analysis in the vagal dissociated populations indeed shows that AITC-evoked responses in the tdT+ AITC-sensitive neurons are greater than those of tdT- AITC-sensitive neurons as anticipated by the reviewer. We agree with the reviewer that the data could be explained by dissociation causing a decrease in tdTomato expression rather than an increase in de novo TRPA1 expression. We now explicitly state that 40% of dissociated vagal neurons are AITC sensitive, and this is almost identical to the percentage of neurons in sliced vagal ganglia that express tdTomato, arguing against de novo TRPA1 expression. The text has been amended to reflect these changes.

As for the functional responses in the intact vagal ganglia, it is probable that the data set is not fully representative of the total vagal population. The GCaMP6s responses depend on AAV-mediated expression of the calcium sensor which varies from cell to cell. It appears as if in these particular studies, the AITC-sensitive/tdT+ neurons were overly represented in the GCaMP6s-expressing vagal population (∼60% vs the expected 40%). This could be either due to AITC-insensitive/tdT- neurons being less efficiently transfected by the AAV or less sensitive to the KCl (positive control) treatment. Although different dorsal root ganglion afferent subsets have been reported to have differing susceptibility to AAV, we are not aware that this has been systematically described in vagal neurons. Previously we performed an intranodose injection in TRPV1-Cre mice with a combination of AAV9-GFP and AAV9-flex-tdT, which would be expected to express GFP in all transfected neurons and tdTomato in TRPV1+ neurons. In that dataset (see Kim et al, 2020; PMID: 32060036), we found that 70% of GFP+ vagal neurons also expressed tdT (almost no GFP-/tdT+ neurons were observed). This is somewhat higher than the expected percentage of TRPV1-expressing neurons in the intact ganglia (∼45-60%). This suggests a mild (but not excessive) preference for AAV9 (following intranodose injection) to transfect nociceptive neurons in the vagal ganglia, similar to our current data. However, given the apparently mild effect, we are hesitant to raise this possibility without further study.

7. The mismatch between tdTomato staining and AITC sensitivity in thoracic DRG neurons is marked. Only 12.5% of DRG neurons in intact ganglia expressed detectable tdTomato but this was reduced to 5.5% in dissociated cell culture, again suggesting reduced tdTomato expression after dissociation. In contrast, ∼32% of the neurons in culture responded to AITC, which is similar to the percentage of responsive neurons (37%) recorded in intact GCamp6 ganglia. As stated by the authors the low percentage of tdTomato-positive neurons may be due to inefficient tdTomato expression in DRG neurons but the sensitivity required to detect tdTomato fluorescence may also be a significant factor.

Authors’ response: We have clarified in the methods the system that we used to detect tdTomato in these studies. We agree that tdTomato levels in some tdT-expressing neurons may have been too low for detection in the dissociated neurons, particularly in DRG neurons. However, given that 71.1% of DRG tdTomato expression in frozen sections was detectable from the native fluorescence alone, we could predict that improved detection (i.e. using antibodies) might increase the % of tdT+ expression in dissociated DRG cultures to 7.7%. Thus it is likely that the bulk of the mismatch between tdTomato expression and AITC-sensitivity in the dissociated DRG dataset is due to a lack tdTomato in many AITC-sensitive neurons, rather than a failure of tdTomato detection. We have clarified this issue in the discussion.

8. The study also examines tdTomato expression in the CNS and several peripheral tissues where TRPA1 expression has previously been reported. This section of the manuscript raises some other concerns. Studies with control R26ai65f/ai65f mice show that Flp-independent expression of tdTomato can occur in some but not all blood vessels. It is not clear that control R26ai65f/ai65f mice were studied to check whether the tdTomato staining seen in other non-sensory nerve structures in the stomach, colon and cochlea were Flp-dependent or - independent and therefore related to TRPA1 expression. These data should be included. Furthermore, the lack of tdTomato fluorescence in other cells particularly those previously reported to `express TRPA1 could simply reflect undetectable, low levels of tdTomato expression in these cells as found in many DRG neurons.

Authors’ response: We agree that the appropriate R26ai65f/ai65f tissue is necessary for the interpretation. We had included the statement “We noted consistent Flp-independent tdTomato expression in many LE-lectin+ blood vessels within the esophagus (both muscle and mucosal layers), trachea, stomach, colon and bladder of R26ai65f/ai65f mice (Fig. 8A, B and 9A, B; n=4 mice). Immunohistochemical staining with an anti-DSred antibody confirmed the presence of tdTomato in these control mice (not shown). tdT+ blood vessels were only occasionally observed in the skin and were absent from the lungs and cochlea (not shown)” in the results. Thus we had presented esophageal, stomach and colon tissue from these control mice in Fig. 8A and 9A, and these images showed that some blood vessels in these organs expressed low levels of tdTomato. Importantly, these images also showed that tdTomato expression in nerve terminals and the stellate-shaped resident cells were absent in R26ai65f/ai65f tissue and thus dependent on the Flp allele associated with the TRPA1 gene. We have now included cochlea data for the control mice. We agree that the lack of A1-flp-tdTomato efficiency observed in the DRG could explain the lack of tdTomato expression in specific cell types in this model and had made this argument in the 2nd last paragraph in the discussion.

9. In the discussion, the authors suggest some reasons for the failure to detect tdTomato fluorescence in every cell. Studies with the second reporter line generated (TRPA1Flp/+R26FLTG/+) demonstrate that this is not dependent on the Flp-sensitive reporter. The authors speculate that higher expression levels may be attained by using homozygous TRPA1FLP mice or by using Cre rather than Flp reporter mice. The sensitivity required to detect the fluorescence should also be discussed. The antibody staining study with DRG neurons showed that tdTomato expression is not always evident from the native dtTomato fluorescence.

Authors’ response: We agree that the antibody detection of tdTomato allowed for more reporter+ neurons to be determined, and we have reiterated this important point in the discussion.

Minor Points:

1. Animal Use: The authors mention that both male and female mice were used for the experiments. Please clarify if they were homogenously distributed and used in all the experiments. Were the slices / cells a pool or combination from male and female?

Authors’ response: This is correct. This has been clarified in the methods.

2. The in vivo experiment analyzing the nocifencive response elicited by chemogenetic activation of TRPA1-expressing nerves the number of mice per group vary between 3 and 6. Please, indicate if they were male and/or female and how many times the experiment was performed (Fig. 12).

Authors’ response: We have included the sex distribution of the mice in the nocifensive studies and clarified that these were paired studies.

3. Review subject-verb agreement: Line 205 (trachea were instead of was); Line 276 (Changes... was instead of were); Line 352 (time... were instead of was)...

Authors’ response: Done

4. Line 149: Substitute Glaborus by glabrous.

Authors’ response: Done

5. Line 154: The trachea, esophagus and... (add “lungs”)

Authors’ response: The lung dissection is described in a later section. The bladder dissection has been added to the sentence identified by the reviewer.
